# Virulence Gene Profiling and Pathogenicity Characterization of Non-Typhoidal *Salmonella* Accounted for Invasive Disease in Humans

**DOI:** 10.1371/journal.pone.0058449

**Published:** 2013-03-07

**Authors:** Jotham Suez, Steffen Porwollik, Amir Dagan, Alex Marzel, Yosef Ilan Schorr, Prerak T. Desai, Vered Agmon, Michael McClelland, Galia Rahav, Ohad Gal-Mor

**Affiliations:** 1 The Infectious Diseases Research Laboratory, Sheba Medical Center, Tel-Hashomer, Israel; 2 Sackler Faculty of Medicine, Tel Aviv University, Tel Aviv, Israel; 3 The Vaccine Research Institute of San Diego, San Diego, California, United States of America; 4 Government Central Laboratories, Ministry of Health, Jerusalem, Israel; 5 Department of Pathology and Laboratory Medicine, University of California Irvine, Irvine, California, United States of America; Facultad de Medicina, Uruguay

## Abstract

Human infection with non-typhoidal *Salmonella* serovars (NTS) infrequently causes invasive systemic disease and bacteremia. To understand better the nature of invasive NTS (iNTS), we studied the gene content and the pathogenicity of bacteremic strains from twelve serovars (Typhimurium, Enteritidis, Choleraesuis, Dublin, Virchow, Newport, Bredeney, Heidelberg, Montevideo, Schwarzengrund, 9,12:l,v:- and Hadar). Comparative genomic hybridization using a *Salmonella enterica* microarray revealed a core of 3233 genes present in all of the iNTS strains, which include the *Salmonella* pathogenicity islands 1–5, 9, 13, 14; five fimbrial operons (*bcf, csg, stb, sth*, *sti*); three colonization factors *(misL, bapA, sinH*); and the invasion gene, *pagN*. In the iNTS variable genome, we identified 16 novel genomic islets; various NTS virulence factors; and six typhoid-associated virulence genes (*tcfA*, *cdtB*, *hlyE*, *taiA*, STY1413, STY1360), displaying a wider distribution among NTS than was previously known. Characterization of the bacteremic strains in C3H/HeN mice showed clear differences in disease manifestation. Previously unreported characterization of serovars Schwarzengrund, 9,12:l,v:-, Bredeney and Virchow in the mouse model showed low ability to elicit systemic disease, but a profound and elongated shedding of serovars Schwarzengrund and 9,12:l,v:- (as well as Enteritidis and Heidelberg) due to chronic infection of the mouse. Phenotypic comparison in macrophages and epithelial cell lines demonstrated a remarkable intra-serovar variation, but also showed that *S*. Typhimurium bacteremic strains tend to present lower intracellular growth than gastroenteritis isolates. Collectively, our data demonstrated a common core of virulence genes, which might be required for invasive salmonellosis, but also an impressive degree of genetic and phenotypic heterogeneity, highlighting that bacteremia is a complex phenotype, which cannot be attributed merely to an enhanced invasion or intracellular growth of a particular strain.

## Introduction

*Salmonella enterica* is a Gram-negative, facultative intracellular human and animal pathogen posing a major public health concern worldwide [1]. The single species *S*. *enterica* includes more than 2,600 serovars, which are taxonomically classified into six subspecies, sharing high sequence similarity [2]. Subspecies I serovars, adapted for mammals and avian hosts, are responsible for more than 99% of all *Salmonella* infections in humans. These serovars can be divided into two clinically relevant groups according to the disease they cause. Infections with the human-restricted serovars *S*. Typhi and *S*. Paratyphi elicit an invasive, life-threatening systemic disease referred to as typhoid or enteric fever [3]. On the other hand, non-typhoidal serovars (NTS) will normally cause in humans, self-limited gastroenteritis, associated with intestinal inflammation and diarrhea [4]. Nevertheless, in developed countries up to 5% of NTS cases may be invasive, extra-intestinal disease leading to bacteremia and focal systemic infections. Moreover, in Sub-Saharan Africa invasive non-typhoidal *Salmonella* (iNTS) have emerged as a major cause of bloodstream infection in adults and children, with an estimated annual incidence of 175–388 cases per 100 000 children and 2000–7500 cases per 100 000 HIV-infected adults [5].

Different factors including the genetic background and the immunological status of the host are now known to predispose to iNTS disease and were recently reviewed [6]. Nevertheless, since certain NTS serovars such as *S*. Choleraesuis and *S*. Dublin are inclined to cause bacteremia more frequently than others [7], it is clear that pathogen characteristics are greatly important as well. Indeed, the unique genetic features of a dominant African invasive genotype of *S*. Typhimurium ST313, which was shown to comprise a distinct prophage repertoire, unique drug resistant elements, and evidence for genome degradation [8], further emphasize the contribution of pathogen genetics to the invasive outcome.

Genomic diversity across bacterial strains is largely shaped by gain of functions via horizontal gene transfer [9]. Foreign acquired DNA containing functionally related genes is designated Genomic Island or Islet (GI). GIs that encode virulence genes are referred to as Pathogenicity Islands (PIs) and their presence in the genome of bacterial pathogens distinguish them from closely related nonpathogenic strains or species (reviewed in [10]).

In the species *S. enterica*, 21 *Salmonella* Pathogenicity Islands (SPIs) have been identified thus far [11], in addition to the *Salmonella* genomic island 1 (SGI-1) [12] and the high-pathogenicity island (HPI) [13]. SPIs are considered to be ‘quantum leaps’ in the evolution of *Salmonella* [14], playing a fundamental role in pathogenesis [15] and host specificity [16]. While certain SPIs (such as SPI-1 and SPI-2) have been studied in depth, other SPIs have been identified more recently and much less is known about their distribution across *Salmonella* serovars and the role they play in disease.

Characterization of the mechanisms underlying invasive manifestation by NTS is essential to a more general understanding of the biology and pathogenicity of *Salmonella*. To better understand the genetic profile and the virulence of iNTS strains, we determined the virulence gene content in *S*. Typhimurium invasive and gastroenteritis strains and in invasive isolates from eleven additional serovars. Furthermore, the pathogenicity of invasive isolates representing the 12 serovars was characterized in the mouse model and virulence-associated phenotypes were compared between invasive and gastroenteritis isolates in tissue cultures models *in-vitro*.

## Materials and Methods

### Bacterial Strains and Growth Conditions

Bacterial strains utilized in this study are listed in Table S1 and include low-passage clinical strains isolated from blood (N = 66), stool (N = 68) or unknown source (N = 1) from 12 NTS serovars as well as *Salmonella* reference sequenced strains (N = 9). All clinical isolates were obtained from the Israeli National *Salmonella* Reference Center after serotyping according to the White-Kauffmann-LeMinor scheme by agglutination with O- and H-antigen specific sera. All *Salmonella* reference strains were purchased from the *Salmonella* Genetic Stock Center (SGSC) at the University of Calgary. Bacterial cultures were routinely maintained in Luria-Bertani (LB; BD Difco) liquid medium at 37°C. LB or xylose lysine deoxycholate (XLD; BD Difco) agar plates were used when appropriate. The clinical isolates that were analyzed by CGH were selected based on patient’s age information and included strains isolated from 2<patients <60 year-old to minimize age-related bias.

### Comparative Genomic Hybridization (CGH)

Genomic DNA from *S*. Typhimurium LT2 and clinical strains was extracted from overnight cultures grown in LB using the GenElute kit (Sigma-Aldrich) according to manufacturer's instructions. DNA labeling and hybridization to the STv7E *Salmonella* microarray (http://www.sdibr.org/Faculty/mcclelland/mcclelland-lab/mcclelland-protocols) were performed as previously described [17]. An Agilent microarray scanner G2505B was used for image acquisition and signal intensities were quantified with the Spotreader software (Niles Scientific). Data normalization, analysis, and determining the presence or absence of genes, were described elsewhere [17].

### Phylogenetic Analysis

A maximum parsimony tree was constructed according to the presence-absence data obtained by the CGH of the clinical isolates and discrete data that were inferred from the genome sequences of reference taxa (in total 5375 genes × 46 taxa were analyzed). The tree was obtained using the Close-Neighbor-Interchange algorithm with search level 1, in which the initial trees were obtained with the random addition of positions (5 replicates). Branch lengths were calculated using the average pathway method to represent the number of changes. The bootstrap consensus tree inferred from 100 replicates, and the evolutionary analyses were conducted in MEGA5.

### PCR and Southern Blot Hybridization

Primers used in this study are listed in Table S2. DNA primers were purchased from IDT and PCR was carried out using ReddyMix PCR (Thermo Scientific) or PfuUltra II Fusion HS DNA Polymerase (Stratagene). For Southern blot analysis, 1 µg of genomic DNA was digested at 37°C for 16–18 h with *Pst*I, subjected to electrophoresis in 1.0% agarose gels before being capillary transferred and cross-linked onto Hybond-N membranes (Amersham Biosciences). Genomic DNA from *E. coli* DH5α was included as a negative control in all hybridizations. *S*. Typhi CT18, *S*. Typhimurium DT104, *S*. Typhimurium 14028 s or SARC13 were used as positive controls. Southern blots were processed using the DIG DNA Labeling and Detection Kit (Roche Applied Sciences), followed by an anti-DIG detection according to the manufacturer’s protocol.

### RT-PCR

RNA was extracted from *Salmonella* cultures that were subcultured into fresh LB broth and grown aerobically to late-exponential-phase using the Qiagen RNAprotect Bacteria Reagent and the RNeasy mini kit (Qiagen) according to the manufacturer’s instructions, including an on-column DNase digest. Purified RNA was secondarily treated with an RNase-free DNase I followed by ethanol-precipitation and 150 ng of DNase I-treated RNA was subjected to a first strand cDNA synthesis, using the iScript cDNA synthesis kit (Bio-Rad Laboratories) according to the kit protocol. 2 µl from the reverse transcription reaction were used as a template for a PCR amplification using the primers listed in Table S2.

### Tissue Culture Conditions and Bacterial Infection

The human epithelial HeLa and the murine macrophages-like RAW264.7 cell-lines were purchased from ATCC and cultured in a high glucose (4.5 g/L) Dulbecco’s Modified Eagle Medium (DMEM) supplemented with 10% heat-inactivated fetal bovine serum (FBS), 1 mM pyruvate and 2 mM L-Glutamine. All cell-lines were cultured at 37°C in a humidified atmosphere with 5% CO_2_. Epithelial cells and RAW264.7 macrophages were seeded at 5×10^4^ and 2.5×10^5^ cells/ml, respectively in a 24-well tissue culture dish 18 to 24 h prior to bacterial infection and experiments were carried out using the gentamicin protection assay as was previously described [18]. HeLa cells were infected at multiplicity of infection (MOI) of ∼1∶100 with *Salmonella* strains that had been subcultured from an overnight culture and grown for 3 h to late logarithmic phase under aerobic conditions. RAW264.7 cells were infected at MOI of ∼1∶1 using overnight stationary grown cultures. At the desired time points post infection (p.i.), cells were washed three times with Phosphate Buffered Saline (PBS) and harvested by addition of lysis buffer (0.1% SDS, 1% Triton X-100 in PBS). Appropriate dilutions were plated on LB-agar plates for bacterial enumeration by CFU count. *Salmonella* invasion was determined by the number of intracellular *Salmonella* at 2 h p.i. divided by the number of infecting bacteria. Survival and intracellular multiplication (in macrophages and in non-phagocytic cells, respectively) were determined by the number of recovered intracellular *Salmonella* at 24 h p.i. divided by the number of intracellular *Salmonella* at 2 h p.i. An unpaired t test with two tails was used to determine the significance of the differences between the compared data and p<0.05 was considered to be statistically significant. All isolates analyzed in TC were examined and found to be gentamicin sensitive and lysis buffer resistant.

### Virulence Experiments in Mice

Six-weeks-old female C3H/HeN and BALB/c mice were purchased from Harlan laboratories and housed at the Sheba Medical Center animal facility under specific pathogen free conditions. All the mice were kept in sterilized cages and given food and water *ad libitum*. The mice were monitored daily, and any that showed extreme distress or became moribund were sacrificed. Experiments in this study were approved and carried out according to the national animal care guidelines and the institutional ethic committee of the Sheba Medical Center (approval No 601/10). Groups of 4–6 mice were infected intraperitoneally (i.p.) with ∼5×10^3^ CFU in 0.3 ml saline. Fecal pellets and organs harvested at end-points were homogenized in cold saline, diluted and plated on selective XLD-agar plates (which distinguish the black *Salmonella* colonies) for CFU enumeration. An unpaired t test with two tails was used to determine the significance of the differences between the compared data and p<0.05 was considered to be statistically significant.

## Results and Discussion

### Core and Variable Genome of iNTS

Bacteremia and systemic infection challenge the pathogen with distinct environmental conditions and stresses, different from the ones in the gastrointestinal ambient [19]. It is therefore expected that a defined set of genes including specific virulence factors will be required and positively selected during this particular lifestyle. To better understand the genetic profile of iNTS and reveal their virulence factor composition we have analyzed the gene content of bacteremic and gastroenteritis *S*. Typhimurium isolates (five from each group) in addition to bacteremic strains from eleven other NTS serovars (Enteritidis, Choleraesuis, Dublin, Virchow, Newport, Bredeney, Heidelberg, Montevideo, Schwarzengrund, 9,12:l,v:- and Hadar). These serovars were chosen to provide a global view onto the iNTS phenomenon and were of the leading cause of NTS bacteremia in Israel, collectively responsible for over 85% of NTS invasive cases during 1995–2011. All of the invasive isolates analyzed in this study accounted for systemic disease in humans and were isolated from patients’ blood as listed in Table S1.

**Figure 1:**
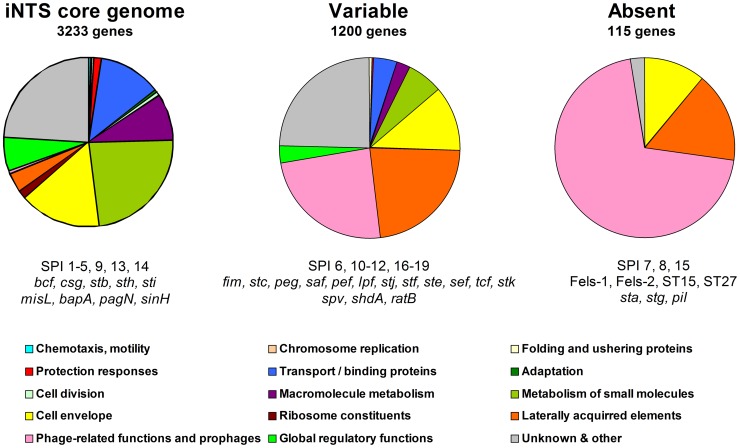
Core, variable and absent genes in iNTS strains. Microarray-based comparative genomic hybridization was used to determine the presence and absence of the *Salmonella* ORFs represented on the *S. enterica* STv7E microarray. After excluding redundant probes (corresponding to the same ORF) and ambiguous predictions, we were able to assign 4548 *Salmonella* genes into three groups. A set of 3233 common genes was determined to be present in all of the bacteremic strains and defined as the core genome of iNTS (left panel). 1200 genes with varying distribution were missing from, at least, one serovar and were considered as the variable iNTS genome (center panel). 115 genes were absent from all the 16 blood strains analyzed in this study (right panel). Functional classification of the genes is shown and assigned using GenProtEC (http://genprotec.mbl.edu/).

CGH approach using the *S. enterica* STv7E microarray was applied to determine on a single-gene resolution the distribution of 5648 *Salmonella* ORFs represented on the array. The STv7E microarray contains nearly all (>96%) genes encoded in *S*. Typhimurium LT2, *S*. Typhimurium SL1344, *S*. Typhi TY2, *S*. Typhi CT18, *S*. Paratyphi A SARB42, *S*. Enteritidis PT4 and the LT2 pSLT virulence plasmid. To verify some of the microarray results and to explore the presence of certain genetics elements, not represented on the array (such as the Gifsy-3 and the ST64B bacteriophages, HPI-1 and SGI-1) we also used Southern blot hybridizations and PCR of selected targets. Overall, from a total of 5656 *Salmonella* ORFs examined by all methods (CGH, PCR and southern blot), 3233 ORFs (57%) were shared by all 16 bacteremic isolates from 12 serovars and therefore considered as the “core” genome of the iNTS strains (Fig. 1). This pool of common genes includes besides many ancestral housekeeping ORFs, horizontally transferred genes encoded within SPIs 1–5, 9, 13 and 14; five fimbrial clusters (*bcf, csg, stb, sth* and *sti*); the SPI-6 encoded invasin, *pagN*; and the CS54 invasin, *sinH*. Although these genes can be found also in reference or gastroenteritis isolates (Table S3), their common presence in all of the invasive isolates (from 12 serovars) suggests that they might be universally required by NTS serovars for invasive manifestation in the human host.

By contrast, 1200 genes were found to be absent (or had no close homologue) in at least one invasive isolate, demonstrating a large degree of genetic diversity among these bacteremic strains (that displayed the same invasive phenotype). The accessory genome included many mobile elements-encoded genes such as the virulence plasmid and prophages, but also multiple metabolic operons, colonization factors and known virulence genes (Fig. 1). In addition, we identified 16 previously uncharacterized genomic islets composed of 2–8 ORFs presenting inconsistent distribution among the NTS serovars (Text S1 and Table S4). Sporadic and patchy distribution indicates either ancestral acquisition following by loss in multiple subsequent lineages, or independent gaining by lateral transfer at different stages during *Salmonella* evolution. Both scenarios elucidate the dynamics and plasticity of the NTS genome.

115 ORFs were found to be entirely absent from all of the iNTS strains and are mainly *S*. Typhi, *S*. Paratyphi, Fels-1, and Fels-2 genes (Fig. 1).

The identified core genome is very different from a previously reported core genome of *Salmonella enterica* subspecies I [20] (see Table S3) and may reflect both technical (e.g. usage of a pan-*Salmonell*a vs. *S*. Typhi CT18 arrays) and biological (e.g. choice of serovars and isolates) differences. Highlighted list, presenting the distribution of 200 virulence-associated genes is shown in Table 1 and the complete data set, including comparison to sequenced reference genomes can be viewed in Table S3.

**Table 1:** Distribution of virulence genes across NTS bacteremic isolates.

Virulence loci	Gene	Locus tag	Typhimurium (Stool)	Typhimurium (Blood)	Schwarzengrund	9,12:l,v:-	Bredeney	Choleraesuis	Dublin	Enteritidis	Hadar	Heidelberg	Montevideo	Newport	Virchow
SPI-1	*sitA*	STM2861	+	+	+	+	+	+	+	+	+	+	+	+	+
	*avrA*	STM2865	+	+	+	−	+	−	+	+	+	+	−	+	+
	*sprB*	STM2866	+	+	+	+	+	+	+	+	+	+	+	+	+
	*orgA*	STM2869	+	+	+	+	+	+	+	+	+	+	+	+	+
	*prgH*	STM2874	+	+	+	+	+	+	+	+	+	+	+	+	+
	*hilD*	STM2875	+	+	+	+	+	+	+	+	+	+	+	+	+
	*hilA*	STM2876	+	+	+	+	+	+	+	+	+	+	+	+	+
	*iagB*	STM2877	+	+	+	+	+	+	+	+	+	+	+	+	+
	*sptP*	STM2878	+	+	+	+	+	+	+	+	+	+	+	+	+
	*sipA*	STM2882	+	+	+	+	+	+	+	+	+	+	+	+	+
	*sipD*	STM2883	+	+	+	+	+	+	+	+	+	+	+	+	+
	*sipC*	STM2884	+	+	+	+	+	+	+	+	+	+	+	+	+
	*sipB*	STM2885	+	+	+	+	+	+	+	+	+	+	+	+	+
	*spaS*	STM2887	+	+	+	+	+	+	+	+	+	+	+	+	+
	*spaN*	STM2892	+	+	+	+	+	+	+	+	+	+	+	+	+
	*invI*	STM2893	+	+	+	+	+	+	+	+	+	+	+	+	+
	*invA*	STM2896	+	+	+	+	+	+	+	+	+	+	+	+	+
		STM2901	+	+	+	+	+	−	−	−	+	+	+	+	+
		STM2902	+	+	+	+	+	ND	−	−	+	+	+	+	+
		STM2905	+	+	−	−	−	+	+	+	+	+	+	+	+
SPI-2	*ttrC*	STM1384	+	+	+	+	+	+	+	+	+	+	+	+	+
	*ssrB*	STM1391	+	+	+	+	+	+	+	+	+	+	+	+	+
	*ssaB*	STM1393	+	+	+	+	+	+	+	+	+	+	+	+	+
	*spiA*	STM1394	+	+	+	+	+	+	+	+	+	+	+	+	+
	*sseC*	STM1400	+	+	+	+	ND	+	+	+	+	+	+	+	ND
	*sseF*	STM1404	+	+	+	+	+	ND	+	+	+	+	+	+	+
	*sseG*	STM1405	+	+	+	+	+	+	+	+	+	+	+	+	+
	*ssaQ*	STM1418	+	+	+	+	+	+	+	+	+	+	+	+	+
SPI-3		STM3752	+	+	−	+	−	−	+	+	−	+	−	+	−
	*sugR*	STM3753	+	+	−	+	−	−	+	+	−	+	−	+	−
		STM3754	+	+	−	+	−	−	+	+	−	+	−	−	−
	*rhuM*	STM3755	+	+	−	+	−	−	+	+	+	+	−	−	−
	*misL*	STM3757	+	+	+	+	+	+	+	+	+	+	+	+	+
	*marT*	STM3759.S	+	+	+	+	+	+	+	+	+	+	+	+	+
	*mgtC*	STM3764	+	+	+	+	+	+	+	+	+	+	+	+	+
		STM3780	+	+	+	+	+	+	−	−	+	+	+	−	+
SPI-4	*siiD*	STM4260	+	+	+	+	+	+	+	+	+	+	+	+	+
	*siiE*	STM4261	+	+	+	+	+	+	+	+	+	+	+	+	+
	*siiF*	STM4262	+	+	+	+	+	+	+	+	+	+	+	+	+
SPI-5	*pipA*	STM1087	+	+	+	ND	+	+	+	+	+	+	+	+	+
	*pipB*	STM1088	+	+	+	+	+	+	+	+	+	+	+	+	+
	*sopB*	STM1091	+	+	+	+	+	+	+	+	+	+	+	+	+
	*pipD*	STM1094	+	+	+	+	+	+	+	+	+	+	+	+	+
SPI-6	*sciL*	STM0277	+	+	+	+	+	−	−	−	+	+	+	+	−
	*sciR*	STM0284	+	+	+	+	+	+	+	−	+	+	+	+	−
	*sciS*	STM0285	+	+	+	+	+	+	+	−	+	+	+	+	−
	*safA*	STM0299	+	+	ND	+	−	+	−	ND	+	−	−	−	−
	*safB*	STM0300	+	+	+	+	+	+	+	+	+	−	+	+	+
	*tcfA^b^*	STY0345	−	−	+	+	+	+	−	−	−	+	+	−	+
	*tinR*	STY0349	−	−	+	+	+	+	−	−	−	+	+	−	+
	*pagN*	STM0306	+	+	+	+	+	+	+	+	+	+	+	+	+
SPI-7	*pilQ*	STY4545	−	−	−	−	−	ND	−	−	−	−	−	−	−
	*pilV*	STY4550	−	−	−	−	−	−	−	−	−	−	−	−	−
	*tviE*	STY4656	−	−	−	−	−	−	ND	−	−	−	−	−	ND
	*vexE*	STY4651	−	−	ND	−	ND	ND	−	−	−	−	−	−	−
	*vexC*	STY4653	−	−	−	−	ND	ND	−	−	−	ND	−	−	−
	*vexA^a^*	STY4655	−	−	−	−	−	−	−	−	−	−	−	−	−
SPI-8		STY3283	−	−	−	−	−	ND	−	−	−	−	−	−	−
		STY3281^a^	−	−	−	−	−	−	−	−	−	−	−	−	−
SPI-9		STY2878	+	+	+	+	+	+	+	+	+	+	+	+	+
	*bapA*	STM2689	+	+	+	+	+	+	+	+	+	+	+	+	+
SPI-10		STY4826	−	−	−	+	−	−	−	−	−	−	−	−	−
	*sefA*	STY4836a	ND	ND	−	−	−	−	+	+	−	−	−	−	−
	*sefD^a^*	STY4839	−	−	−	−	−	−	+	+	−	−	−	−	−
SPI-11	*envF*	STM1240	+	+	−	−	−	+	+	+	+	+	−	+	+
	*pagD*	STM1244	+	+	+	ND	ND	+	+	+	ND	+	+	+	+
	*pagC*	STM1246	+	+	+	+	+	+	+	+	ND	+	+	+	+
	*pltA*	STY1890	−	−	+	+	+	−	−	−	−	−	+	−	−
	*pltB*	STY1891	−	−	+	+	+	−	−	−	−	−	+	−	−
	*cdtB^b^*	STY1886	−	−	+	+	+	−	−	−	−	−	+	−	−
SPI-12	*sspH2*	STM2241			−	−	−	+	+	+	ND	ND	−	ND	ND
	*oafA*	STM2232	+	+	−	−	−	ND	−	−	−	+	−	−	−
SPI-13		STM3118	+	+	+	+	+	+	+	+	+	+	+	+	+
		STM3121	+	+	+	+	+	+	+	+	+	+	+	+	+
SPI-14		STM0854	+	+	+	+	+	+	+	+	+	+	+	+	+
		STM0859	+	+	+	+	+	+	+	+	+	+	+	+	+
SPI-15		STY3188	−	−	−	−	−	−	−	−	−	−	−	−	−
		STY3191^a^	−	−	−	−	−	−	−	−	−	−	−	−	−
SPI-16		STM0557			−	+	−	−	+	+	−	+	−	−	−
	*gtrA*	STM0559			+	+	+	ND	+	+	+	+	+	+	+
SPI-17		STY2629^a^	−	−	−	−	−	−	+	+	−	−	−	−	−
		STY2631			−	−	−	ND	+	+	ND	ND	−	ND	ND
SPI-18	*hlyE^b^*	STY1498	−	−	+	+	+	−	−	−	−	−	+	−	−
	*taiA*	STY1499	−	−	+	+	+	−	−	−	−	−	+	−	−
SPI-19	*impA*	SEN1003	−	−	−	−	−	−	+	+	−	−	−	−	−
	*icmF*	SEN1004	−	−	ND	ND	−	ND	+	+	−	−	ND	−	−
CS54	*xseA*	STM2512	+	+	+	+	+	+	+	+	+	+	+	+	+
	*shdA*	STM2513	+	+	−	−	−	+	+	+	+	+	−	+	+
	*ratB*	STM2514	+	+	−	−	−	+	+	+	−	+	−	+	−
	*ratA*	STM2515	+	+	+	+	+	+	+	+	+	+	+	+	+
	*sinI*	STM2516	+	+	+	ND	ND	+	+	+	+	ND	+	+	+
	*sinH*	STM2517	+	+	+	+	+	+	+	+	+	+	+	+	+
Gifsy-1	*gipA*	STM2600		4/5	−	−	−	+	−	−	+	−	−	−	+
	*gogB*	STM2584		4/5	−	−	−	+	−	−	−	−	−	−	−
Gifsy-2	*sodC1*	STM1044		4/5	−	−	−	+	+	+	−	+	−	+	−
	*gvrA*	STM1034		4/5	−	−	−	ND	+	−	−	+	−	+	−
	*sseI*	STM1051		4/5	−	−	−	+	+	+	ND	ND	−	ND	ND
	*gtgE*	STM1055		4/5	−	−	−	+	+	+	−	ND	−	−	−
Gifsy-3	*sspH1^b^*		−	−	−	−	−	−	−	−	−	−	−	−	−
Fels-1	*nanH*	STM0928	−	−	−	−	−	−	−	−	−	−	−	−	−
	*sodCIII*	STM0924	−	−	−	−	−	−	−	−	−	−	−	−	−
Fels-2		STM2695	−	−	−	−	−	−	+	−	−	−	−	−	−
		STM2723	−	−	−	−	−	−	−	−	−	−	−	−	−
		STM2739	−	−	−	−	−	−	+	−	−	−	−	−	−
Def4		STM4196	+	+	−	−	−	+	+	+	−	+	−	−	+
		STM4198													
		STM4205													
		STM4213													
ST64b	*Sb46^a^*			4/5	−	−	−	+	+	+	−	+	−	−	−
	*ssek3^b^*		3/5	1/5	−	−	−	−	+	+	−	−	−	−	−
SopEФ		STY4619	−	−	−	−	−	−	−	−	−	−	−	−	−
	*sopEФ^a,b^*	STY4609		1/5	−	−	−		+	+	+	+	−	+	+
*S.* Typhi phage ST10		STY1046	3/5	3/5	ND			ND							
		STY1048		1/5				+							
		STY1054		1/5				+							
		STY1071		2/5				+							
*S.* Typhi phage ST18		STY2019		2/5				+							
		STY2028		2/5				+							
		STY2036		1/5				+							
ST15	*gam*	STY1601	−	−	−	−	−	−	−	−	−	−	−	−	−
ST27		STY2885	−	−	−	−	−	−	−	−	−	−	−	−	−
ST35	*cI*	STY3660			−	−	−	−	−	−	−	−	−	−	−
	*nucE*	STY3681	−	−	−	−	−	−	+	−	−	−	−	−	−
	*nucD*	STY3682	−	−	−	−	−	−	+	−	−	−	−	−	−
		STY3693	−	−	−	−	−	−	−	−	−	−	+	−	−
ST46		STY4826	−	−	−	+	−	−	−	−	−	−	−	−	−
SPA-1		SPA2388	4/5	4/5	−	+	+	+	+	−	−	+	+	−	+
	*sieB*	SPA2416	4/5	4/5	−	+	+	−	−	−	−	+	+	−	−
	*ral*	SPA2418	2/5	2/5	−	−	−	−	−	−	−	+	−	−	ND
SPA-3		SPA2613	−	−	−	+	−	−	−	−	−	−	−	−	−
pSLT	*rck*	PSLT009	+	+	ND	−	ND	ND	ND	+	−	−	ND	−	−
	*pefD*	PSLT016		4/5	−	−	−	+	−	+	−	−	−	−	−
	*pefA*	PSLT018		4/5	−	−	−	−	−	−	−	−	−	−	−
	*spvC*	PSLT038	+	+	−	−	−	+	+	+	−	−	−	−	−
	*spvB*	PSLT039	+	+	ND	ND	−	+	+	+	−	−	ND	−	−
	*spvA*	PSLT040	+	+	−	−	−	+	+	+	−	−	−	−	−
	*spvR*	PSLT041	+	+	−	−	ND	+	+	+	−	ND	−	−	−
	*mig*-*5*	PSLT046	+	+	−	−	−	+	+	+	−	−	−	−	−
HPI-1	*fyuA^a^*		−	−	−	−	−	−	−	−	−	−	−	−	−
SGI-1	*S023^a^*		2/5	2/5	−	−	−	−	−	−	−	−	−	−	−
	*S024^a^*		2/5	2/5	−	−	−	−	−	−	−	−	−	−	−
other fimbriae & adhesins	*staE*	STY0203	−	−	−	−	−	−	−	−	−	−	−	−	−
	*steA*	STY3084	+	+	+	+	+	+	+	+	+	+	+	+	+
	*steB*	STY3086	−	−	−	−	−	+	+	+	+	+	−	+	+
	*stgB*	STY3919	−	−	−	−	−	−	−	−	−	−	−	−	−
	*bcfA*	STM0021	+	+	+	+	+	+	+	+	+	+	+	+	+
	*fimA*	STM0543	+	+	+	+	+	+	+	+	+	+	+	+	+
	*fimH*	STM0547	+	+	+	+	+	−	+	+	+	+	+	+	+
	*lpfC*	STM3638	+	+	−	−	−	+	+	+	+	+	−	+	+
	*csgA*	STM1144	+	+	+	+	+	+	+	+	+	+	+	+	+
	*stbC*	STM0338	+	+	+	+	+	+	+	+	+	+	+	+	+
	*stcB*	STM2151	+	+	−	−	−	+	−	−	+	+	−	+	+
	*pegD*	SEN2144A	−	−	+	+	+	−	+	+	−	−	−	−	−
	*stfD*	STM0197	+	+	−	−	−	+	+	+	+	+	−	+	+
	*sthD*	STM4592	+	+	+	+	+	+	+	+	+	+	+	+	+
	*stiC*	STM0175	+	+	+	+	+	+	+	+	+	+	+	+	+
	*stjC*	STM4573	+	+	−	+	−	−	−	−	+	+	+	+	+
	*stkE*	SPA0181	−	−	−	−	−	−	−	−	+	+	−	−	+
	*stdD*	STM3026	+	+	−	−	−	−	+	+	−	+	−	−	−
	*stdC*	STM3027	+	+	+	+	+	+	+	+	+	+	+	+	+
regulators	*zur*	STM4241	+	+	+	+	+	+	+	+	+	+	+	+	+
	*hfq*	STM4361	+	+	+	+	+	+	+	ND	+	+	+	ND	ND
	*igeR*	STM0410	+	+	−	−	−	+	+	+	+	+	−	+	+
	*relA*	STM2956	+	+	+	+	+	+	+	+	+	+	+	+	+
	*phoQ*	STM1230	+	+	+	+	+	+	+	+	+	+	+	+	+
	*phoP*	STM1231	+	+	+	+	+	+	+	+	+	+	+	+	+
	*fur*	STM0693	+	+	+	+	+	+	+	+	+	+	+	+	+
	*hns*	STM1751	+	+	+	+	+	+	+	+	+	+	+	+	+
	*hnr*	STM1753	+	+	+	+	+	+	+	+	+	+	+	+	+
	*rpoE*	STM2640	+	+	+	+	+	+	+	+	+	+	+	+	+
	*rpoS*	STM2924	+	+	+	+	+	+	+	+	+	+	+	+	+
	*oxyR*	STM4125	+	+	+	+	+	+	+	+	+	+	+	+	+
	*slyA*	STM1444	+	+	+	+	+	+	+	+	+	+	+	+	+
	*ompR*	STM3502	+	+	+	+	+	+	+	+	+	+	+	+	+
	*rtcR*	STM3522	+	+	−	−	−	+	+	+	−	+	−	−	−
	*sirA*	STM1947	+	+	+	+	+	+	+	+	+	+	+	+	+
Effectors outside of SPIS and other virulence factors	*virK*	STM2781	+	+	+	+	+	+	+	+	+	+	+	+	+
	*mig*-*14*	STM2782	+	+	+	+	+	+	+	+	+	+	+	+	+
	*slrP*	STM0800	+	+	+	+	+	+	+	+	+	+	+	+	+
	*zirT*	STM1668	+	+	ND	−	−	+	+	+	+	−	ND	+	+
	*zirS*	STM1669	+	+	+	+	+	+	+	+	+	+	+	+	+
	*sopA*	STM2066	+	+	+	+	+	+	+	+	+	+	+	+	+
	*sopE2*	STM1855	+	+	+	+	+	+	+	+	+	+	+	+	+
	*sopD*	STM2945	+	+	+	+	+	+	+	+	+	+	+	+	+
	*sopD2*	STM0972	+	+	+	+	+	+	+	+	+	+	+	+	+
	*sifA*	STM1224		+	+	ND	ND	+	+	+	+	+	+	+	+
	*sifB*	STM1602	+	+	+	+	+	+	+	+	+	+	+	+	+
	*sseJ*	STM1631	+	+	+	+	ND	+	+	+	+	+	+	+	+
	*sseK1*	STM4157	+	+	−	+	ND	+	+	+	+	−	+	−	+
	*sseK2*	STM2137	+	+	ND	ND	ND	+	ND	ND	+	+	−	+	ND
	*msgA*	STM1241	+	+	+	+	+	+	+	+	+	+	+	+	+
	*tolC*	STM3186	+	+	+	+	+	+	+	+	+	+	+	+	+
	*iroN*	STM2777	+	+	+	+	+	+	+	+	+	+	+	+	+
	*pipB2*	STM2780	+	+	+	+	ND	+	+	+	+	+	+	+	+
	*sseL*	STM2287	+	+	+	+	+	+	+	+	+	+	+	+	+
	*srfJ*	STM4426	+	+	−	−	−	−	−	−	+	−	−	−	+
	*steA*	STM1583	+	+	+	+	+	ND	+	+	+	+	+	+	+
	*steB*	STM1629	+	+	+	+	+	+	+	+	+	+	+	+	+
	*steC*	STM1698	+	+	+	+	+	+	+	+	+	+	+	+	+
		STY1413	−	−	+	+	+	−	−	−	−	−	+	−	−
		STY1360	−	−	+	+	+	−	−	−	−	−	+	−	−

The presence-absence of genes associated with *Salmonella* virulence were determined in five *S*. Typhimurium stool isolates (115043, 88359, 93561, 98001, 130100) and five blood isolates (103259, 111682, 116449, 93130, 98666) in addition to 11 invasive strains from serovars Schwarzengrund (124983), 9,12:l,v:- (94293), Bredeney (96115), Choleraesuis (90958), Dublin (74007), Enteritidis (122205), Hadar (121851), Heidelberg (78646), Montevideo (111072), Newport (91532) and Virchow (103033). Where not stated otherwise, presence was determined by CGH using the *Salmonella* STv7E microarray. *a*, presence was determined or confirmed by southern blot; *b*, presence was determined or confirmed by PCR. A plus sign indicates the presence of the gene; white blocks indicate an absence; a variable presence among the blood or stool isolates of *S*. Typhimurium is shown by the number of positive isolates/ total number of isolates (N = 5); ND, no data (CGH was not conclusive).

Additionally, intra-serovar comparison between invasive (N = 5) and gastroenteritis (N = 5) *S*. Typhimurium isolates revealed 127 ORFs that were found to be conserved in the invasive strains, but variable among the examined stool isolates (Table S5). These genes included the *pef* (plasmid-encoded fimbriae) operon; the Gifsy-1 encoded effector, *gogB*; the Gifsy-2 encoded effector, *sseI*; the periplasmic [Cu,Zn]-superoxide dismutase, *sodC*; an attachment/invasion protein (STM1043) and the region spanning STM2740-STM2771 containing two sugar phosphotransferase systems (STM2750-2752 and STM3256-3260). To get a further insight into their distribution we examined their presence in a larger collection of 15 invasive and 15 enteritis *S*. Typhimurium isolates (including the isolates analyzed by CGH). PCR analysis showed that *pefA*, *SodC* and *gogB* were variably present in both invasive and enteritis isolates, while *sseI*, STM2759 (as a probe for the STM2740-STM2771 region), and STM3260 (*gatC*) were found in all of the invasive isolates (15/15) and in 14/15 of the enteritis strains (Table S6). Although these results confirmed the variable presence among *S*. Typhimurium isolates, they did not indicate a clear origin-related distribution for any of the aforementioned genes.

### Distribution of SPIs and type Three Secretion System (T3SS) Effectors among iNTS

Many of the virulence factors of *S*. *enterica* are encoded by genes organized on SPIs that have been acquired by horizontal transfer and believed to be self-mobile [21]. The overall distribution of the SPIs identified in the iNTS strains could be divided into three categories: (i) intact (or mostly intact) SPIs with universal presence in all of the iNTS isolates (SPIs 1–5, 9, 13 and 14); (ii) SPIs that are completely absent from this iNTS collection (SPIs 7, 8 and 15); and (iii) SPIs with variable or mosaic presence across the serovars (SPIs 6, 10–12 and 16–19).

Interestingly, SPI-18 which was considered to be *S*. Typhi-specific [11], was found to be present in several NTS serovars (see below). Also, SPI-17 that was described before in *S*. Typhi [22], *S*. Enteritidis, *S*. Gallinarum [23] and *S*. Typhimurium LT2 [24] was now identified in *S*. Dublin as well. The *Yersinia* high pathogenicity island (HPI) that was recently found in *S*. Senftenberg poultry isolates [13] was not present in any of the tested iNTS isolates; however, the SGI-1 [25] was identified in two invasive (116449 and 98666) and two gastroenteritis (93561 and 115043) *S*. Typhimurium isolates.

With the exception of *avrA* that was found to be variable across isolates, other SPI-1, SPI-2, and SPI-5 encoded effector genes were conserved in their presence. The SPI-1 and SPI-2 T3SSs-substrate gene, *sspH1*, was absent from all examined isolates and at least ten other T3SS effector genes (all encoded outside of SPI-1, SPI-2 and SPI-5) were variably distributed, including *sspH2, sseK1, sseK2, srfJ*, and the prophages encoded effectors: *gogB, sseI, gipA, gtgE*, *sseK3* and s*opE*. Many of these effectors including SopE were shown to play a role in *Salmonella* virulence (reviewed in [26]); however their absence from multiple iNTS isolates suggests that they are unnecessary for invasive manifestation in the human host.

SopE translocation into cultured cells leads to actin cytoskeletal rearrangements and membrane ruffling [27] and many of the *S*. Typhimurium epidemic strains identified so far were reported to carry *sopE* [28]. To learn more about *sopE* distribution among bacteremic strains we screened by PCR a larger collection of 35 clinical isolates from blood and stool sources representing the above 12 serovars (Table S1). These analyses indicated the absence of *sopE* from 4/5 of the blood isolates of serovars Typhimurium, Schwarzengrund (3 isolates) and from at least one isolate of serovar Enteritidis (isolate 10325; data not shown), supporting the notion that SopE is nonessential for invasive salmonellosis in humans.

### Distribution of Fimbriae and Colonization Factors

*S*. *enterica* subspecies I serovars harbors a wide array of putative fimbriae and pili central to bacterial adherence [29]. *Salmonella* serovars were shown to harbor a unique combination of fimbrial genes, believed to play a role in host tropism and colonization [30]. Among 20 fimbrial operons examined, a core set of 5 operons (*bcf, csg, stb, sth* and *sti*) seemed to be conserved in all bacteremic isolates, whereas three operons (*stg*, *sta* and *pil*), considered as “typhoid” fimbriae were missing from all serovars. The *peg* fimbrial cluster (SEN2144A-SEN2145B) that was first identified in *S*. Enteritidis PT4 [23] and was recently shown to be required for systemic infection of this serovar in mice [31], was revealed, for the first time, also in serovars Bredeney, 9,12:l,v:-, Dublin and Schwarzengrund (Table 1).

Besides fimbriae, additional colonization factors including the SPI-3 encoded adhesin, *misL*; the SPI-4 encoded adhesin, *siiE*; the SPI-6 encoded invasin, *pagN*; the SPI-9 encoded biofilm-associated protein, *bapA*; and the CS54 invasin, *sinH* were all present through the entire iNTS collection, implying that they may play a role in an invasive lifestyle of NTS, possibly by contributing to tissue tropism and/or to niche-specificity.

### Presence and Expression of Typhoid-associated Virulence Factors in NTS Serovars

CGH analysis identified a curious presence of several virulence factors that were originally characterized in *S*. Typhi and are believed to play a role in enteric fever manifestation. One of these factors is the cytolethal distending toxin CdtB, shown to be expressed by intracellular *S*. Typhi and cause cell-cycle arrest, severe host cell distension and enlargement of the nucleus [32]. In *S*. Typhi, *cdtB* (STY1886) is encoded within SPI-11 together with *pltA* (STY1890) and *pltB* (STY1891) that were shown to form an intracellular tripartite toxin that is transported to the cell surface via a vesicular mechanism [33]. CGH revealed the presence of *cdtB*, *pltA* and *pltB* in isolates of four NTS serovars including Montevideo, Schwarzengrund, Bredeney and 9,12:l,v:- (Table 1).

**Figure 2:**
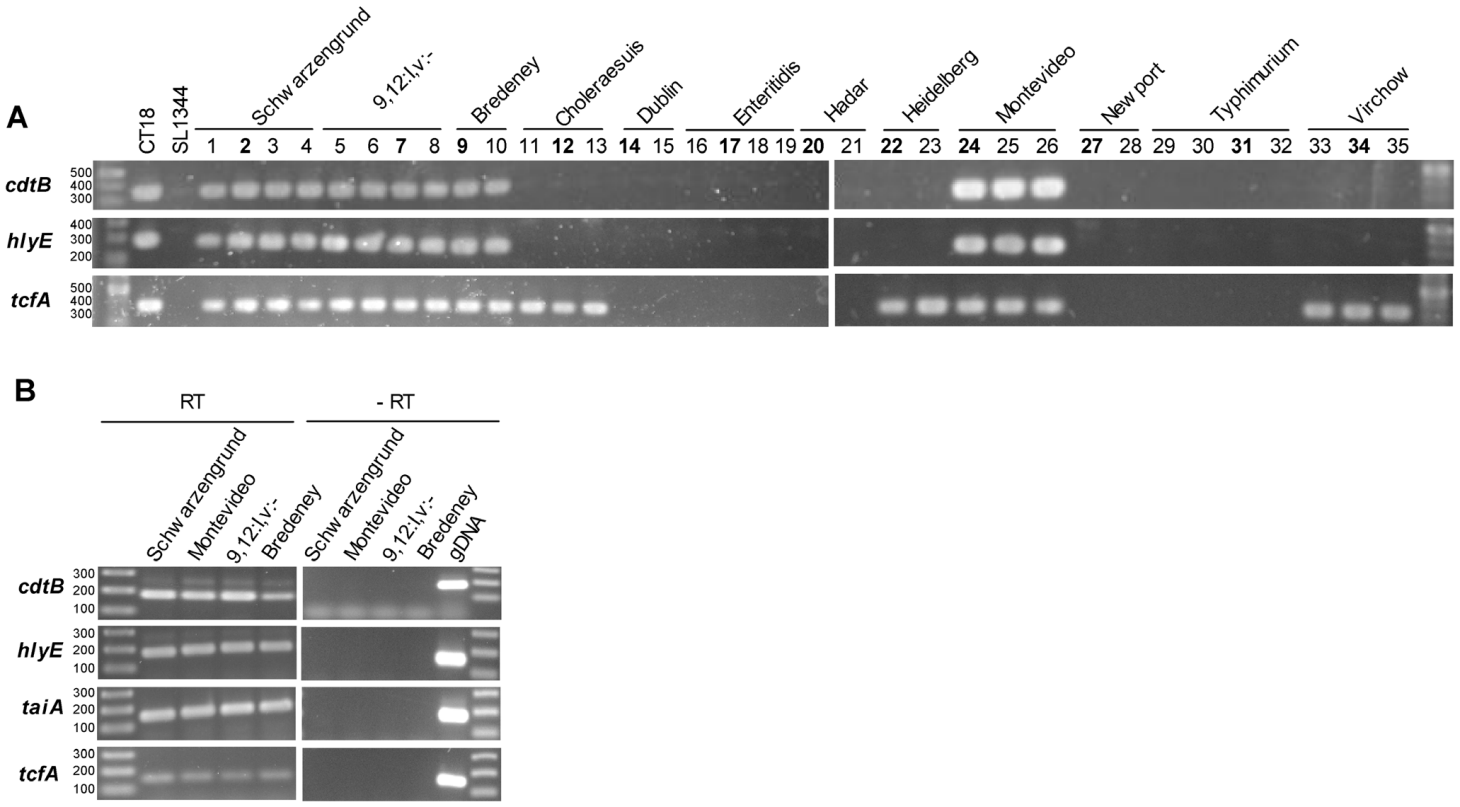
Presence and expression of typhoid-associated virulence factors among NTS serovars. (A) PCR analysis was used to confirm CGH results and to determine the distribution of three typhoid-associated genes in 35 clinical isolates from the 12 NTS serovars. PCR amplicons of 353-bp, 294-bp, and 335-bp indicate the presence of *cdtB*, *hlyE* and *tcfA*, respectively. Tested isolates are numbered from 1–35 according to Table S1, with the isolates that were characterized in mice and subjected to CGH analysis in bold. *S.* Typhi CT18 (CT18) and *S.* Typhimurium SL1344 (SL1344) were used as positive and negative controls, respectively. (B) Reverse transcription-PCR was applied to examine expression of *cdtB*, *hlyE, taiA* and *tcfA* genes in serovars Schwarzengrund, Montevideo, 9,12:l,v:- and Bredeney. Bacterial RNA was extracted from *Salmonella* cultures grown to late logarithmic phase in LB, followed by treatment with DNase I and reverse transcription. cDNA was used as template for PCR amplification using the primers listed in Table S2. *Salmonella* RNA without a reverse transcriptase treatment (-RT) and purified gDNA were used as negative and positive controls, respectively.

To broaden and verify the above CGH results we expanded the number of tested strains and examined by PCR 35 clinical isolates (Table S1). This analysis confirmed the array results and indicated the presence of *cdtB* in 13/13 clinical strains from serovars Schwarzengrund, 9,12:l,v:-, Bredeney and Montevideo (Fig. 2A).

Another *S*. Typhi toxin is the pore-forming hemolysin HlyE (ClyA) that accumulates in the periplasm of *S*. Typhi [34]. *hlyE* (STY1498) is encoded within SPI-18 together with the Typhi-associated invasin A (*taiA*, STY1499), and is required for efficient invasion of human epithelial cells. Functional transfer of *S.* Typhi *hlyE* to *S*. Typhimurium was shown to improve colonization of deep organs (spleen, liver) in mice [35], [36]. TaiA is a secreted, 27 kDa invasin that increases *Salmonella* uptake by human macrophages and is controlled by the virulence-related regulator PhoP [35]. Like in the case of *cdtB*, CGH indicated the presence of SPI-18 in the invasive isolates of Schwarzengrund, Montevideo, 9,12:l,v:- and Bredeney (Table 1 and Fig. 2A).

These results are in line with a recent report by den Bekker *et al*. [37], showing the presence of *cdtB* and *hlyE-taiA* in *S*. Schwarzengrund and Montevideo isolates, but also reveal their presence, for the first time, in serovars 9,12:l,v:- and Bredeney.

*S*. Typhi carries a fimbriae cluster known as the Typhi-colonization factor (*tcf*) operon (STY0345- STY0348) encoded in SPI-6 and was suggested to play a role in host specificity of *S*. Typhi to humans [38]. In addition to Typhi and Paratyphi A, the *tcf* operon was found present in serovars Choleraesuis, Schwarzengrund and Heidelberg [29], [39] and recently also in Virchow and Montevideo [37]. Our results confirmed the presence of *tcf* in blood isolates of these serovars and further identified its previously unknown presence in 9,12;l,v;- and Bredeney (Table 1 and Fig. 2A).

After finding the presence of *cdtB, hlyE and tcfA* in clinical isolates of serovars Schwarzengrund, 9,12:l,v:-, Bredeney and Montevideo, we have further examined a larger collection of 40 invasive and 43 gastroenteritis isolates from these four serovars (including the relevant 13 isolates shown in Fig. 2). The results presented in Table S7 showed that while *tcfA* was found in 3/4 invasive isolates of *S*. Schwarzengrund (as well as in the two reference sequenced genomes CVM19633 and SL480) it was uncommonly found in only 1/12 gastroenteritis isolates; indicating a variable and possibly source-related distribution of *tcfA* in *S*. Schwarzengrund. Yet, *tcfA* was found in all of the isolates from serovars 9,12:l,v:- (15 invasive and 13 gastroenteritis), Bredeney (13 invasive and 12 gastroenteritis) and Montevideo (8 invasive and 6 gastroenteritis). The distribution of *cdtB* and *hlyE* was more unified and found in all of the isolates examined (Table S7), indicating that their presence is typical to, at least, clinical isolates, from serovars 9,12:l,v:-, Bredeney and Montevideo and is not associated with a specific source (i.e. invasive vs. gastroenteritis).

The presence of, so called, “typhoid-virulence genes” in a subset of NTS serovars was interesting and prompted us to test if they are also expressed. RNA purified from the invasive isolates of serovars Schwarzengrund, Montevideo, 9,12:l,v:- and Bredeney was used for a reverse-transcription PCR analysis. The results presented in Fig. 2B demonstrated that *tcfA, hlyE, taiA* and *cdtB* are readily expressed in these four serovars *in-vitro*, under conditions known to induce *Salmonella* invasion [40].

Moreover, homologs of two other yet uncharacterized typhoid genes, STY1413 and STY1360 were also identified in serovars Schwarzengrund, Montevideo, 9,12:l,v:- and Bredeney (Table 1). These two genes are characterized by a distinct G+C content and present amino acid sequence similarity to the EspN2-2 (EHU09761; E-value 6e-72) and EspS (YP_003363979; E-value 5e-14), T3SS effectors of *E. coli* and *Citrobacter rodentium*, respectively, implying that they might function as secreted effector proteins in these *Salmonella* serovars.

Collectively, these results revealed an unknown presence of several typhoid virulence genes in serovars Schwarzengrund, Montevideo, 9,12:l,v:- and Bredeney, establishing that the distribution of these factors outside of typhoidal serovars is wider than previously known. Furthermore, we demonstrated, for the first time, native expression of *cdt*B, and *taiA* in NTS serovars, and presented the first evidence for variable presence of *tcf*A in *S*. Schwarzengrund and for its native expression in serovars 9,12:l,v:- and Bredeney; suggesting that their role is not limited to enteric fever caused by Typhi and Paratyphi. Nevertheless, the absence of these factors from other iNTS isolates indicates that their function is still not universally required by all invasive Salmonellae and cannot explain invasive manifestation by other strains.

### Phylogenetic Clustering of the iNTS Strains

**Figure 3:**
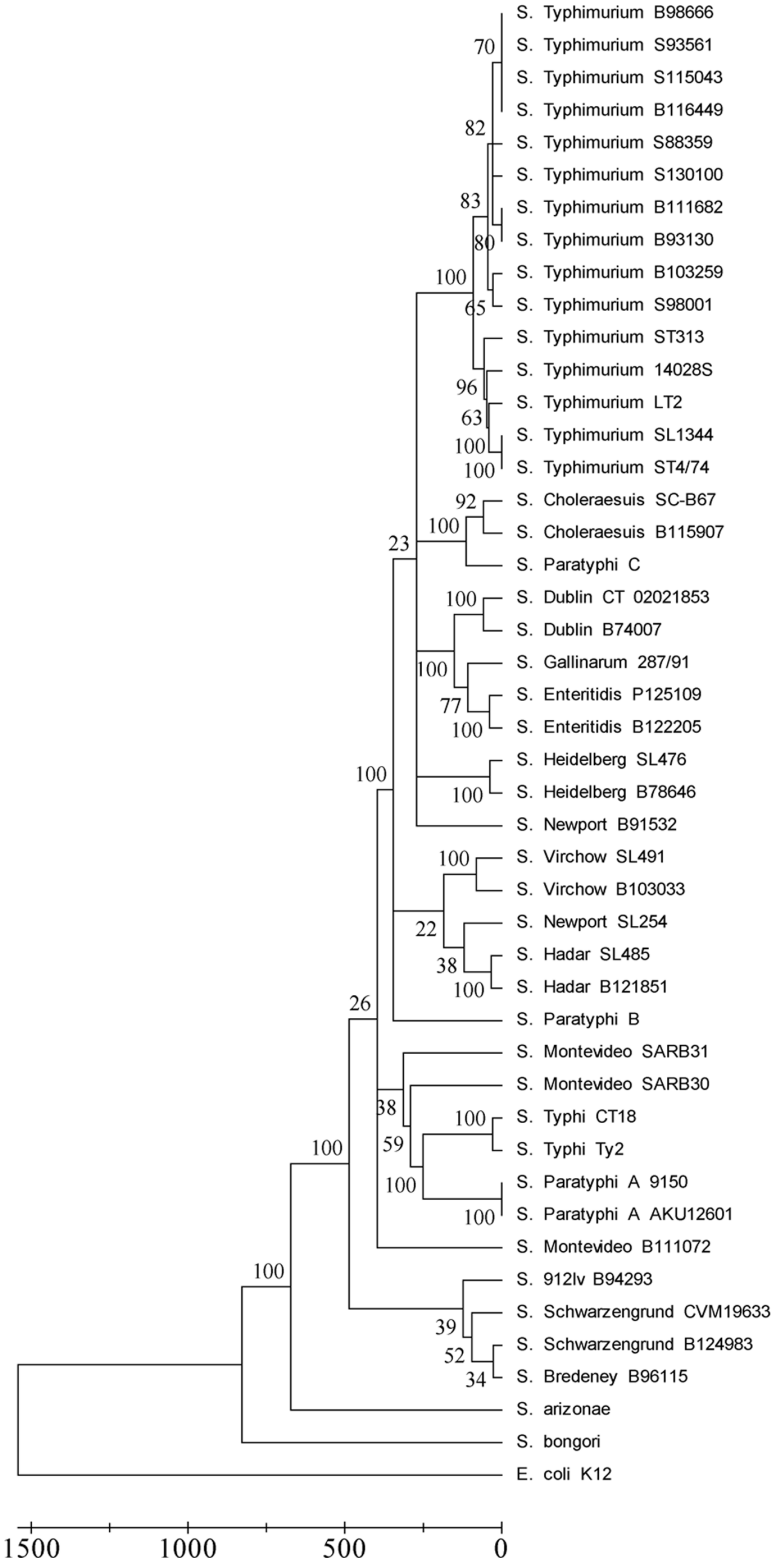
Phylogenetic relationships of the clinical isolates and reference strains. A maximum parsimony tree was constructed according to the presence-absence data obtained by the CGH of the 21 NTS isolates and discrete data that were inferred from the genome sequences of reference taxa (in total 46 taxa × 5374 genes were analyzed). The bootstrap consensus tree inferred from 100 replicates was taken to represent the evolutionary history of the taxa analyzed. The percentage of replicate trees, in which the associated taxa clustered together in the bootstrap test (100 replicates) are shown next to the branches. The tree is drawn to scale, with branch lengths indicating the number of changes. Evolutionary analyses were conducted in MEGA5. Blood isolates are indicated by a leading “B” before the isolate number and stool isolates are marked with a leading “S”.

Data obtained from the CGH (5374 positions) were used to determine the phylogenetic relationship of the various clinical isolates and corresponding reference sequenced strains (46 taxa in total). In particular, we were interested to see how invasive isolates will be clustered in relation to reference genomes and among themselves. A maximum parsimony tree clustered most of the bacteremic isolates together with their corresponding reference genomes and with the clinical gastroenteritis isolates (in the case of *S*. Typhimurium, Fig. 3) and did not group together invasive isolates from different serovars. These results suggested that genetic variation was kept among different bacteremic strains, although displaying the same invasive phenotype (and not a genetic convergence towards a uniform gene content).

### Pathogenicity of iNTS Strains *in-vivo*

**Figure 4:**
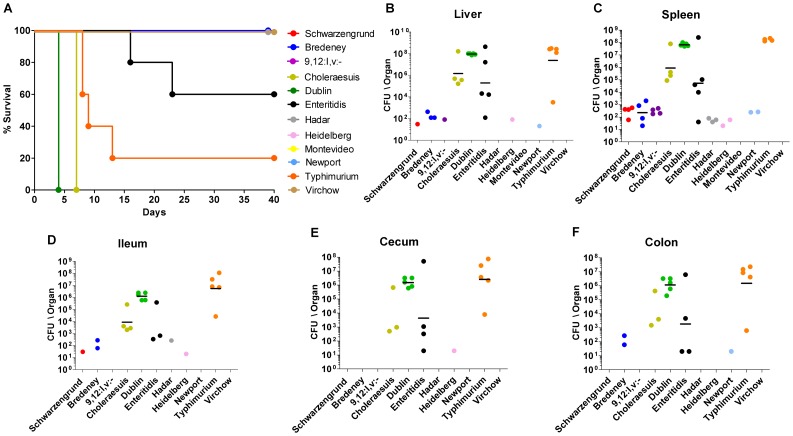
Pathogenicity of iNTS strains in mice. Twelve groups of 4–5 female C3H\HeN mice were challenged i.p. with ∼5×10^3^ CFU of bacteremic strains from serovars Schwarzengrund (isolate number 124983), 9,12:l,v:- (94293), Bredeney (96115), Choleraesuis (90958), Dublin (74007), Enteritidis (122205), Hadar (121851), Heidelberg (78646), Montevideo (111072), Newport (91532), Typhimurium (103259), and Virchow (103033). Survival of the mice during 40 days post-infection is shown (A). At end-points (as shown in A), harvested organs were homogenized and serial dilutions were plated onto XLD-agar plates for CFU count. Bacterial load in each mouse is represented as CFU/organ by individual dots in the liver (B), spleen (C), ileum (D), cecum (E), and colon (F). Geometric mean for each serovar in the different sites is shown by a horizontal line.

Next, we were interested in comparing the virulence of the bacteremic strains in a murine host and asked whether these isolates will present a comparable virulence phenotype. The pathogenicity of 12 bacteremic isolates (that were subjected to CGH analysis) was characterized in C3H/HeN (Nramp1+/+) inbreed mice, by comparing animals survival, colonization in systemic and intestinal sites, and bacterial shedding. The results of an i.p. infection indicated profound differences between the serovars in all of the three examined parameters. Clearly, only isolates, from serovars Typhimurium, Dublin and Choleraesuis, and to a lesser extent, Enteritidis were able to establish an acute systemic infection in this mouse strain (Fig. 4), indicating that a former systemic colonization in human patients did not restrict their host-tropism and the known potential of these serovars to cause systemic infection in mice [41]–[43] was maintained. Serovars Dublin and Choleraesuis killed all the mice in 4 and 7 days, respectively; serovars Typhimurium and Enteritidis killed 80% and 40% of the mice, respectively; while serovars Schwarzengrund, 9,12:l,v:-, Bredeney, Hadar, Heidelberg, Montevideo, Newport and Virchow did not elicit any animal mortality (Fig. 4A). Not surprisingly, infection with the four serovars, which caused mortality (Dublin, Choleraesuis, Typhimurium and Enteritidis), was characterized by relatively high bacterial load in systemic sites (Fig. 4B–C) and in the intestinal tract (Fig. 4D–F).

**Figure 5:**
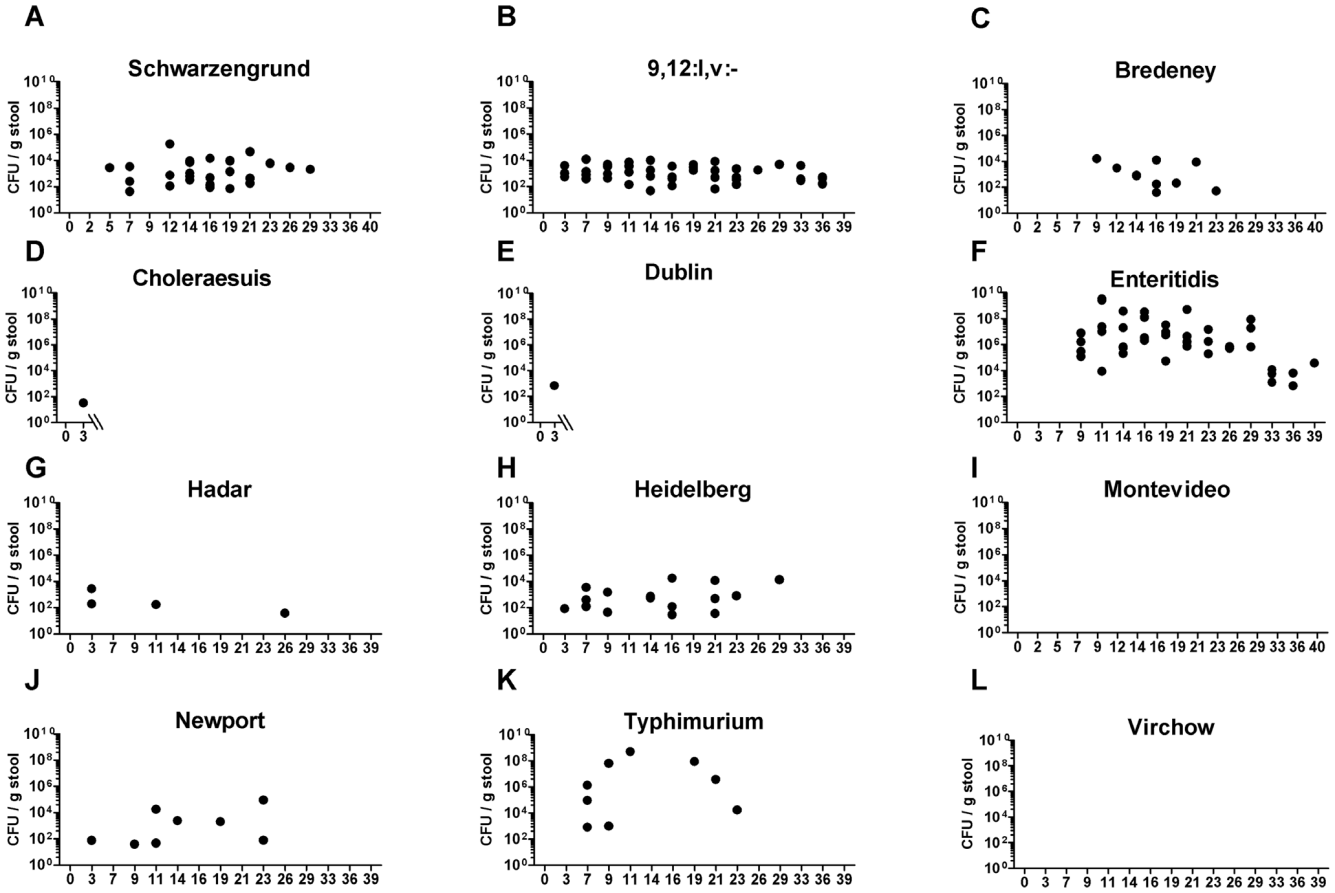
Persistent infection of iNTS strains in the mouse model. Following challenge with ∼5×10^3^ CFU of the bacteremic strains, fecal pellets were collected from the C3H\HeN mice at the indicated time points during 40 days (or until the animal was sacrificed). Pellets were weighed, homogenized in PBS and plated onto XLD-agar plates to determine the number of CFU/g stool. Shedding of *S.* Choleraesuis (D) and *S.* Dublin (E) is shown only until 3 days p.i., as the mice had to be euthanized. Dots represent independent CFU counts in pellets of individual mice.

Another parameter that was studied is bacterial shedding, which was monitored during 40 days p.i. (or until the animal had to be sacrificed). Notably, shedding was not coincident with the severity of the infection or signs of disease, as serovars Heidelberg, Schwarzengrund and 9,12:l,v:-, which showed only low to mild systemic infection, and Enteritidis caused the most profound and prolonged shedding, during a persistent infection in the mouse (Fig. 5). On the other hand, serovars Montevideo and Virchow could not infect the mice at all and did not provoke any bacterial shedding. To the best of our knowledge, this is the first report describing the pathogenicity of serovars Schwarzengrund, 9,12:l,v:-, Bredeney, and Virchow in the mouse model.

The high level of virulence demonstrated by serovars Typhimurium, Enteritidis, Dublin and Choleraesuis is well consistent with previous studies characterizing their pathogenicity in the mouse model [41]–[43]. Also, the poor ability of serovars Hadar, Heidelberg, Montevideo and Newport to infect mice is in agreement with other reports that showed low CFU counts in systemic sites following infection with these serovars [41]–[47]. These observations suggest that although *Salmonella* may enhance its virulence after passage through its host [48], a former systemic infection in humans did not confer adaptive changes facilitating infection in mice.

Taken together, these experiments established that different serovars known to infect mice [41] retained this ability even after systemic manifestation in humans and that invasive infection of NTS in humans does not lead to phenotypic convergence or to virulence uniformity in the murine host. Interestingly, in addition to an inter-serovar variation in the induction of systemic disease, previously attributed to the presence of the virulence plasmid [45], we demonstrated clear differences in the ability of these clinical strains to promote long-term shedding in mice during persistent infection, with particularly high shedding of serovars Schwarzengrund, 9,12:l,v:- and Enteritidis. For that reason, infection of C3H/HeN mice with these isolates may be used as an informative model to study host-pathogen interactions during chronic *Salmonella* infection.

### Are iNTS Strains more Virulent than Gastrointestinal Isolates?

**Figure 6:**
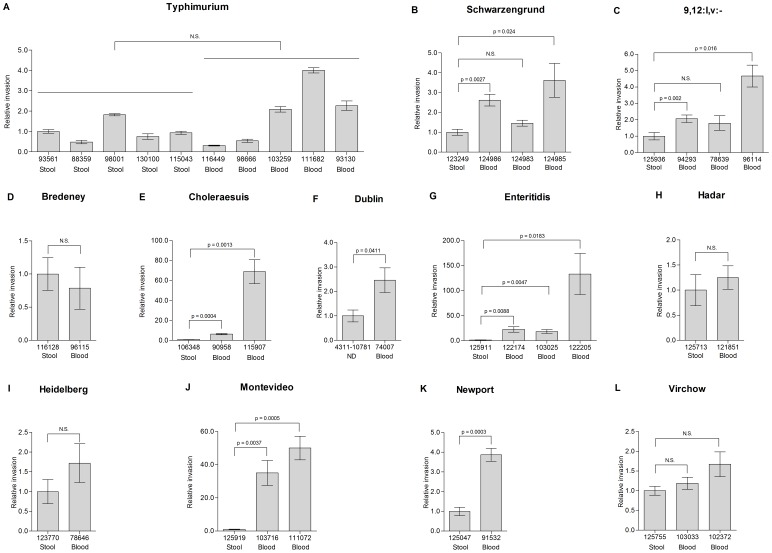
Invasion of bacteremic and enteritis strains into human epithelial cells. Clinical isolates from 12 NTS serovars from blood (invasive) and stool (gastrointestinal) sources were grown to late logarithmic phase in LB medium and used to infect HeLa cells at a MOI of ∼100∶1. The invasion of each strain (source and isolate number are indicated below each bar) is shown in relation to the invasion of the stool isolate in each serovar. In *S*. Typhimurium, invasion is presented relative to median value of the stool isolates (isolate 93561). Indicated values present the mean and the standard error of the mean (SEM; represented by the error bars) of at least 4 independent infections. ND, no data, as the source of isolate 4311–10781 from serovar Dublin is not known.

One possible explanation for *Salmonella* dissemination beyond the intestinal epithelium during salmonellosis is an enhanced virulence capability of the iNTS strains compared to non-invasive isolates. To test this notion, we analyzed epithelial cell invasion, intracellular replication and macrophage survival following infection with clinical isolates from blood (invasive) and stool (gastroenteritis) sources. Invasion experiments using the human epithelial HeLa cell-line showed substantial degree of intra-serovar variation and at least in serovars Typhimurium (Fig. 6A), Schwarzengrund (6B), 9,12:l,v:- (6C), Bredeney (6D), Hadar (6H), Heidelberg (6I) and Virchow (6L) stool isolates were equally (or more) invasive than at least one of the corresponding blood isolates. Only stool isolates from serovars Choleraesuis (6E), Enteritidis (6G), Montevideo (6J) and Newport (6K) were significantly less invasive than the compared blood isolates.

**Figure 7:**
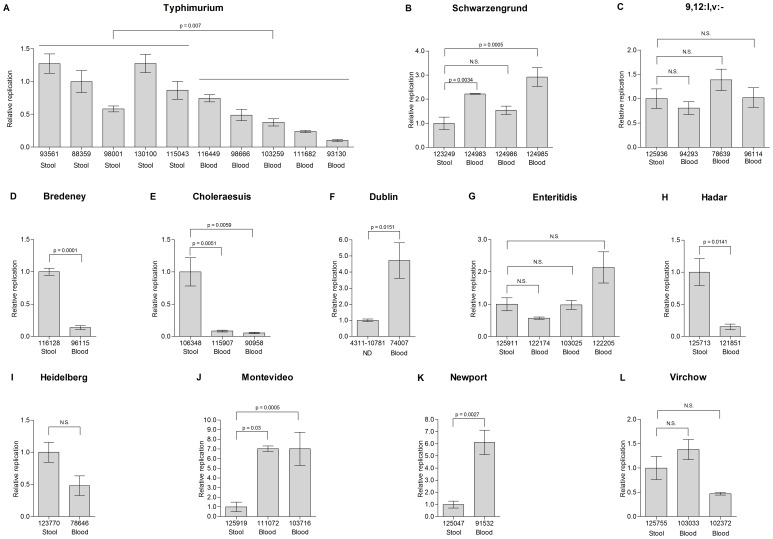
Intracellular growth of invasive and enteritis strains in epithelial cells. Clinical isolates (N = 41) from blood and stool sources were grown to late logarithmic phase in LB medium and used to infect epithelial HeLa cells at a MOI of ∼100∶1. Intracellular replication (ratio between recovered CFU at 24 h/CFU at 2 h p.i.) is shown in relation to the stool isolate of each serovar. In *S*. Typhimurium, replication is presented relative to median value of the stool isolates (isolate 88359). Indicated values present the mean and the SEM of at least 4 independent infections.

Interestingly, when the intracellular replication of *S*. Typhimurium isolates was examined, we found that gastroenteritis isolates tended to present higher intracellular growth than invasive isolates (p = 0.007; Fig. 7A). Higher intracellular replication of gastroenteritis strains compared to the invasive isolates was also observed in serovars Bredeney (7D), Choleraesuis (7E), Hadar (7H) and Heidelberg (7I); while comparable replication was found in serovars Schwarzengrund (Fig. 7B), 9,12:l,v:- (7C), Enteritidis (7G) and Virchow (7L). Only blood isolates from serovars Montevideo (7J) and Newport (7K; 2/12 serovars studied) replicated better than the tested stool isolates. It is therefore tempting to speculate that invasive manifestation might restrain the ability of the pathogen to replicate within epithelial cells, perhaps due to adaptive changes that occurred during the systemic infection.

**Figure 8:**
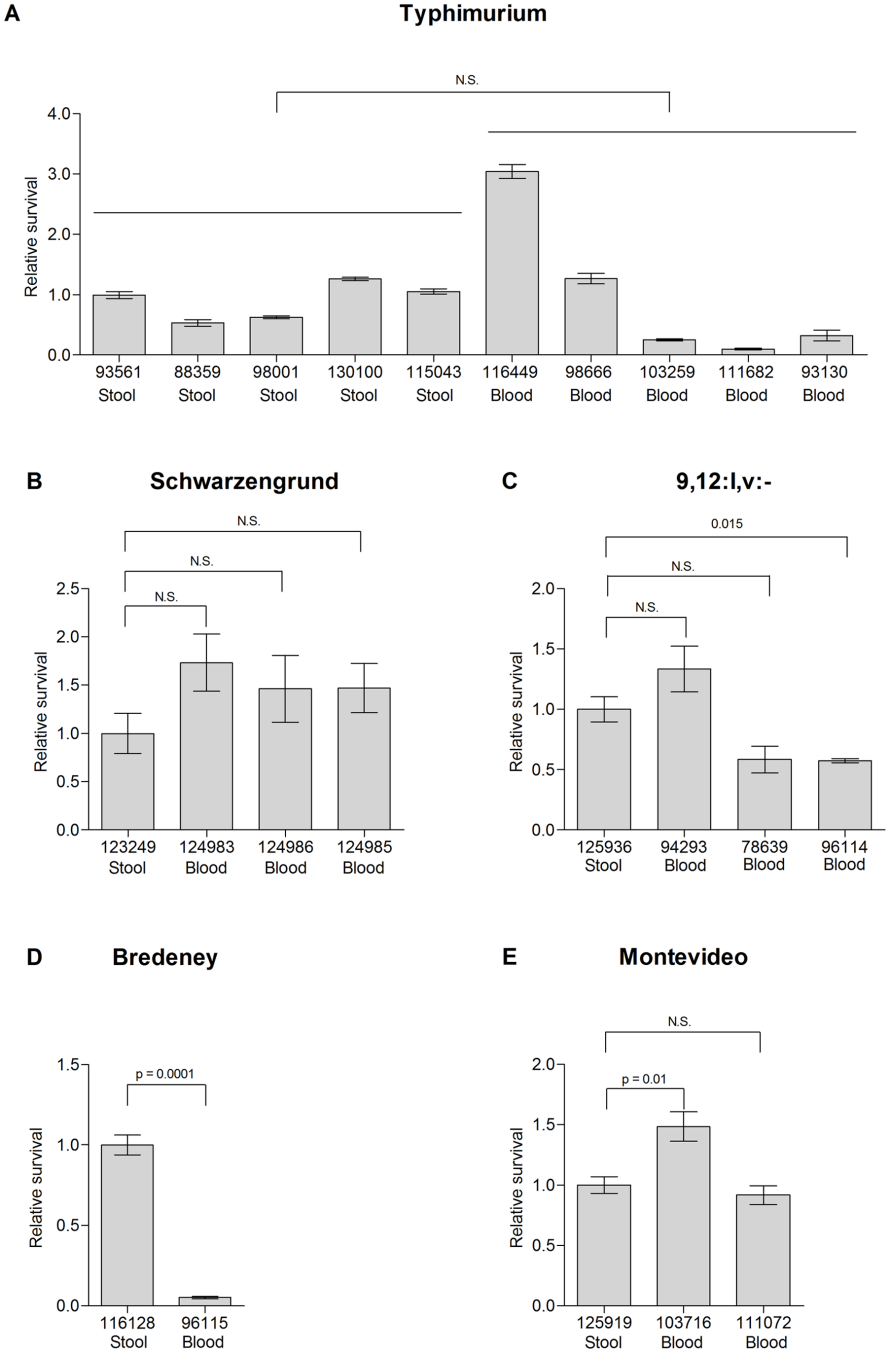
Survival of invasive and enteritis strains in macrophages. *Salmonella* isolates from blood and stool sources from serovars Typhimurium (A), Schwarzengrund (B), 9,12:l,v:-(C), Montevideo (D) and Bredeney (E) were grown to stationary phase in LB medium and used to infect RAW2464.7 cells at a MOI of ∼1. Survival is shown in relation to the stool isolate of each serovar. In *S*. Typhimurium, survival is presented relative to median value of the stool isolates (isolate 93561). Indicated values present the mean and the SEM of at least 4 independent infections.

Intracellular survival of clinical isolates from *S*. Typhimurium and the four serovars found to contain typhoid virulence genes (Fig. 2B) was also tested in macrophage-like RAW 264.7 cells. Again, stool isolates from serovars Typhimurium, Schwarzengrund, 9,12:l,v:-, and Montevideo presented similar macrophage survival to some of the bacteremic strains (Fig. 8 A-D) and the stool isolate of serovar Bredeney survived even better than the blood strain (Fig. 8E). Comparable results were also attained in the mouse model, as blood and stool isolates of serovar Schwarzengrund (124983 and 123249, respectively) reached, 7 days p.i., to similar bacterial loads in the gastrointestinal and systemic sites, following i.p. infection of BALB/c mice (data not shown).

Accumulatively, these experiments indicated a substantial degree of intra-serovar variation in virulence-associated phenotypes, and established that iNTS strains do not generally present superior invasion or macrophage survival *in vitro* and are not hyper-virulent in mice, suggesting that invasive manifestation in humans is not likely due to an elevated virulence potential *per se* of the infected strain.

### Conclusions

To better understand the nature of invasive NTS strains we have characterized the pathogenicity and studied the gene composition of invasive and gastroenteritis isolates from 12 NTS serovars. CGH identified a core genome consisting of SPIs 1–5, 9, 13 and 14; five fimbrial clusters (*bcf, csg, stb, sth*, and *sti*); the SPI-6 encoded invasin, *pagN*; and the CS54 invasin, *sinH* that were common in all of the 16 iNTS strains and may play a role in invasive manifestation of NTS. Nevertheless, many other known *Salmonella* virulence factors, including multiple T3SS effectors, colonization and invasion factors were absent from several bacteremic isolates (such as the lack of SspH1 and SspH2 from serovars Schwarzengrund, 9,12:l,v:- Bredeney, and Montevideo, or the absence of SopE from blood isolates of Typhimurium, Enteritidis and Schwarzengrund), suggesting they are dispensable for invasive infection. Additionally, we revealed an unknown presence of typhoid virulence genes (*tcfA*, *cdtB*, *hlyE*, *taiA*, STY1413, and STY1360) and demonstrated, for the first time, native expression of, *cdt*B, and *taiA* in several NTS serovars, establishing that the distribution, and likely the function, of these factors outside of typhoidal serovars is broader than assumed.

With the possible exception of *tcfA* that was found to be more frequently associated with invasive isolates of *S*. Schwarzengrund than with gastroenteritis isolates, we did not identify a clear source-related distribution of virulence genes. This observation may indicate that potential genetic differences between invasive and gastroenteritis isolates may be more subtle and possibly result from variances that were not analyzed in this study such as genes expression, point mutations or DNA polymorphism.

In the second part of the study we have characterized the pathogenicity of bacteremic strains in an experimental murine infection model and demonstrated a clear variation in disease and in bacterial shedding. We showed that human invasive isolates from serovars Typhimurium, Dublin and Choleraesuis were able to establish an acute systemic infection in C3H/HeN mice, whereas isolates of Schwarzengrund, 9,12:l,v:-, Heidelberg and Enteritidis elicited a persistent infection accompanied by prolonged pathogen shedding. Intra-serovar comparison in epithelial and macrophages cell lines of 41 clinical isolates from the 12 studied serovars exhibited a prominent variation in the virulence-associated phenotypes of isolates from the same serovars and established that blood isolates are not generally more invasive than stool isolates. An intriguing trend of superior intraepithelial cell replication of gastroenteritis isolates compared to invasive isolates was observed in serovars Typhimurium, Bredeney, Choleraesuis, Hadar and Heidelberg, suggesting possible higher intracellular fitness of the former. Collectively, these findings highlight that bacteremia is a complex phenotype, which cannot be explained merely by increased invasion or intracellular growth of a certain strain.

## Supporting Information

Table S1 Bacterial strains used in this study. Laboratory strains and clinical isolates used in the study are listed. Isolate or strain designation, the source and patient age (whom the strain was isolated from) are given. Clinical strains, which were included in the analysis shown in Fig. 2 are numbered 1–35. The specific isolates that were used for the CGH analysis and for mice infection experiments are indicated by the plus (+) sigh. SGSC, *Salmonella* Genetic Stock Center; NA, data is not available.
(DOC)

Table S2 Primers used in this study.
(DOC)

Table S3 Prediction of presence-absence of genes in NTS isolates by CGH. The STv7bE *Salmonella* microarray was used for CGH to determine the presence-absence of 5374 ORFs represented on the array in 16 iNTS isolates and 5 *S*. Typhimurium gastroenteritis isolates. Clinical isolates are shown in grey and reference strains in white. Blood isolates are indicated by the leading “B” and isolate number and stool isolates are indicated with a leading “S” following by the isolate number. Gene tags, symbols and annotations in *S*. Typhimurium (STM), *S*. Typhi (STY), *S*. Paratyphi A (SPA) and *S*. Enteritidis (SEN) are shown. Prediction of present genes is indicated by the number “2” and blue color, while prediction of absent genes is indicated by the number “0” and colored in red. Low signal or ambiguous data (1) are shown in white. The presence-absence of genes in the reference strains was determined using an in-house script that BLAST the DNA sequences of the array probes against the following reference genomes: *E. coli K12* (accession number NC_000913.2), *S. bongori* NCTC12419 (NC_015761.1), *S. arizonae* RSK2980 (NC_010067.1), *S.* Typhi CT18 (NC_003198.1), *S.* Typhi Ty2 (NC_004631.1), *S*. Paratyphi B SPB7 (NC_010102.1), *S*. Paratyphi C RKS4594 (NC_012125.1), *S*. Paratyphi A 9150 (NC_006511.1), *S*. Paratyphi A AKU12601 (NC_011147.1), *S*. Schwarzengrund CVM19633 (NC_011094.1), *S*. Choleraesuis SC-B67 (AE017220.1), *S*. Dublin CT_02021853 (CP001144.1), *S*. Enteritidis P125109 (NC_011294.1), *S*. Gallinarum 287/91 (NC_011274.1), *S*. Hadar SL485 (ABFG00000000), *S*. Heidelberg SL476 (CP001120.1), *S*. Montevideo SARB30 (AESU00000000) and SARB31 (AESR00000000), *S*. Newport SL254 (CP001113.1) and SL317 (ABEW00000000), *S*. Typhimurium LT2 (AE006468.1), SL1344 (FQ312003.1), 14028s (CP001363.1), ST4/74 (NC_016857.1) and ST313 (NC_016854.1) and *S*. Virchow SL491 (ABFH00000000). Data is also compared to a previous report analyzing the core genome of *S. enterica* subs. 1 [20].
(XLS)

Table S4 Novel genomic islets identified in this study. Discrete regions with variable presence among the 12 NTS serovars are listed. Plus (+) indicates the presence of the islets, minus sign (-) indicates its absence and plus-minus sign (±) indicates partial or mosaic presence.
(DOC)

Table S5 Distribution of conserved genes found in five invasive *S*. Typhimurium isolates. *S*. Typhimurium genes, which were found by CGH to be conserved in five invasive isolates (116449, 98666, 103259, 111682 and 93130), but with variable presence in the five gastroenteritis isolates (93561, 88359, 98001, 130100 and 115043) analyzed by CGH are listed. Locus tag, gene symbol and the function of 127 identified genes is indicated. Prediction of present genes among the invasive isolates is indicated by the number “2” and blue color, while prediction of absent genes is indicated by the number “0” and colored in red. Low signal or ambiguous data (1) are shown in white.
(XLS)

Table S6 Distribution of *pefA, sodC, sseI,* STM2759, *gatC* and *gogB* among invasive and enteritis isolates of *S.* Typhimurium. The presence of *pefA, sodC, sseI,* STM 2759, *gatC* and *gogB* was examined by PCR in 15 blood and 15 stool isolates of *S*. Typhimurium. The primers used for this analysis are listed in Table S2. A “+” sign indicates gene presence and “–“ sign indicates its absence.
(DOCX)

Table S7 Distribution of *cdtB, hlyE*, and *tcfA* among invasive and enteritis isolates of serovars Schwarzengrund, 9,12:l,v:-, Bredeney and Montevideo. The presence of *cdtB, hlyE*, and *tcfA* was examined by PCR in clinical isolates of serovars Schwarzengrund, 9,12:l,v:-, Bredeney and Montevideo from blood (invasive) and stool (gastroenteritis) sources. The primers used for this analysis are listed in Table S2. A “+” sign indicates gene presence and “–“ sign indicates its absence.
(DOC)

Text S1 Presence of the *spv* operon and the virulence plasmid; Prophage and bacteriophage remnants; Identification of novel genomics islets.
(DOCX)
