# Supplementary material for: Virulence Gene Profiling and Pathogenicity Characterization of Non-Typhoidal Salmonella Accounted for Invasive Disease in Humans
Source: PLoS One. 2013 Mar 7;8(3):e58449. doi: 10.1371/journal.pone.0058449 (PMC3591323; doi:10.1371/journal.pone.0058449)
Supplement: Table S1 — Bacterial strains used in this study. Laboratory strains and clinical isolates used in the study are listed. Isolate or strain designation, the source and patient age (whom the strain was isolated from) are given. Clinical strains, which were included in the analysis shown in Fig. 2 are numbered 1–35. The specific isolates that were used for the CGH analysis and for mice infection experiments are indicated by the plus (+) sigh. SGSC, Salmonella Genetic Stock Center; NA, data is not available. (DOC) [file pone.0058449.s001.doc]

**Table S1 Bacterial strains used in the study.**

| Species \ Serovar | Isolate \ Strain | Source | Age (y.) | Numbering in Fig. 2 | CGH | Mice infection |
| --- | --- | --- | --- | --- | --- | --- |
| *E. coli* | DH5α | Lab stock | NA |  |  |  |
| *S.* Typhi | CT18 | SGSC | NA |  |  |  |
| 45:g,z(51):(-) | SARC-13 | SGSC | NA |  |  |  |
| *S.* Schwarzengrund | 124986 | Blood | NA | 1 |  |  |
| *S.* Schwarzengrund | 124983 | Blood | NA | 2 | + | + |
| *S.* Schwarzengrund | 124985 | Blood | NA | 3 |  |  |
| *S.* Schwarzengrund | 131982 | Blood | 92 |  |  |  |
| *S.* Schwarzengrund | 123249 | Stool | NA | 4 |  |  |
| *S.* Schwarzengrund | 91899 | Stool | 43 |  |  |  |
| *S.* Schwarzengrund | 93610 | Stool | 75 |  |  |  |
| *S.* Schwarzengrund | 95798 | Stool | 34 |  |  |  |
| *S.* Schwarzengrund | 96750 | Stool | 7 |  |  |  |
| *S.* Schwarzengrund | 109107 | Stool | 4 |  |  |  |
| *S.* Schwarzengrund | 110382 | Stool | 2 |  |  |  |
| *S.* Schwarzengrund | 110460 | Stool | 30 |  |  |  |
| *S.* Schwarzengrund | 112399 | Stool | 7 |  |  |  |
| *S.* Schwarzengrund | 112525 | Stool | 2 |  |  |  |
| *S.* Schwarzengrund | 112900 | Stool | 60 |  |  |  |
| *S.* Schwarzengrund | 118194 | Stool | 18 |  |  |  |
| *S.* Schwarzengrund | CVM19633 | SGSC | NA |  |  |  |
| *S.* 9,12:l,v:- | 78639 | Blood | 2 | 5 |  |  |
| *S.* 9,12:l,v:- | 96114 | Blood | 7 | 6 |  |  |
| *S.* 9,12:l,v:- | 94293 | Blood | 7 | 7 | + | + |
| *S.* 9,12:l,v:- | 92027 | Blood | 92 |  |  |  |
| *S.* 9,12:l,v:- | 92287 | Blood | 1 |  |  |  |
| *S.* 9,12:l,v:- | 96659 | Blood | 75 |  |  |  |
| *S.* 9,12:l,v:- | 99366 | Blood | NA |  |  |  |
| *S.* 9,12:l,v:- | 101823 | Blood | 97 |  |  |  |
| *S.* 9,12:l,v:- | 103439 | Blood | 15 |  |  |  |
| *S.* 9,12:l,v:- | 111027 | Blood | 37 |  |  |  |
| *S.* 9,12:l,v:- | 121750 | Blood | 1 |  |  |  |
| *S.* 9,12:l,v:- | 125271 | Blood | NA |  |  |  |
| *S.* 9,12:l,v:- | 125377 | Blood | NA |  |  |  |
| *S.* 9,12:l,v:- | 133986 | Blood | 39 |  |  |  |
| *S.* 9,12:l,v:- | 134055 | Blood | 19 |  |  |  |
| *S.* 9,12:l,v:- | 125936 | Stool | NA | 8 |  |  |
| *S.* 9,12:l,v:- | 95135 | Stool | 22 |  |  |  |
| *S.* 9,12:l,v:- | 96735 | Stool | 1 |  |  |  |
| *S.* 9,12:l,v:- | 97604 | Stool | <1 |  |  |  |
| *S.* 9,12:l,v:- | 106208 | Stool | <1 |  |  |  |
| *S.* 9,12:l,v:- | 106281 | Stool | <1 |  |  |  |
| *S.* 9,12:l,v:- | 110695 | Stool | 53 |  |  |  |
| *S.* 9,12:l,v:- | 111449 | Stool | 1 |  |  |  |
| *S.* 9,12:l,v:- | 128109 | Stool | <1 |  |  |  |
| *S.* 9,12:l,v:- | 131348 | Stool | <1 |  |  |  |
| *S.* 9,12:l,v:- | 132667 | Stool | 20 |  |  |  |
| *S.* 9,12:l,v:- | 117808 | Stool | 36 |  |  |  |
| *S.* 9,12:l,v:- | 127595 | Stool | 65 |  |  |  |
| *S.* Bredeney | 96115 | Blood | 23 | 9 | + | + |
| *S.* Bredeney | 90321 | Blood | 18 |  |  |  |
| *S.* Bredeney | 92025 | Blood | 66 |  |  |  |
| *S.* Bredeney | 92339 | Blood | 4 |  |  |  |
| *S.* Bredeney | 111361 | Blood | 1 |  |  |  |
| *S.* Bredeney | 116128 | Stool | 1 |  |  |  |
| *S.* Bredeney | 117665 | Blood | 1 |  |  |  |
| *S.* Bredeney | 119214 | Blood | 1 |  |  |  |
| *S.* Bredeney | 123632 | Blood | 1 |  |  |  |
| *S.* Bredeney | 123896 | Blood | 67 |  |  |  |
| *S.* Bredeney | 128407 | Blood | NA |  |  |  |
| *S.* Bredeney | 130875 | Blood | 3 |  |  |  |
| *S.* Bredeney | 133975 | Blood | NA |  |  |  |
| *S.* Bredeney | 134592 | Blood | <1 |  |  |  |
| *S.* Bredeney | 125816 | Stool | 4 | 10 |  |  |
| *S.* Bredeney | 93520 | Stool | 3 |  |  |  |
| *S.* Bredeney | 95097 | Stool | <1 |  |  |  |
| *S.* Bredeney | 97597 | Stool | 69 |  |  |  |
| *S.* Bredeney | 99441 | Stool | 2 |  |  |  |
| *S.* Bredeney | 101983 | Stool | 25 |  |  |  |
| *S.* Bredeney | 113272 | Stool | 1 |  |  |  |
| *S.* Bredeney | 119210 | Stool | 1 |  |  |  |
| *S.* Bredeney | 121976 | Stool | 1 |  |  |  |
| *S.* Bredeney | 122171 | Stool | 71 |  |  |  |
| *S.* Bredeney | 127708 | Stool | 18 |  |  |  |
| *S.* Bredeney | 130076 | Stool | 38 |  |  |  |
| *S.* Bredeney | 131122 | Stool | 2 |  |  |  |
| *S.* Choleraesuis | 115907 | Blood | 2 | 11 |  |  |
| *S.* Choleraesuis | 90958 | Blood | 51 | 12 | + | + |
| *S.* Choleraesuis | 106348 | Stool | NA | 13 |  |  |
| *S.* Dublin | 74007 | Blood | 34 | 14 | + | + |
| *S.* Dublin | 4311-10781 | NA | NA | 15 |  |  |
| *S.* Enteritidis | 122174 | Blood | 52 | 16 |  |  |
| *S.* Enteritidis | 122205 | Blood | 36 | 17 | + | + |
| *S.* Enteritidis | 103025 | Blood | 7 | 18 |  |  |
| *S.* Enteritidis | 125911 | Stool | 2 | 19 |  |  |
| *S.* Hadar | 121851 | Blood | 51 | 20 | + | + |
| *S.* Hadar | 125713 | Stool | <1 | 21 |  |  |
| *S.* Heidelberg | 78646 | Blood | 4 | 22 | + | + |
| *S.* Heidelberg | 123770 | Stool | <1 | 23 |  |  |
| *S.* Montevideo | 111072 | Blood | 3 | 24 | + | + |
| *S.* Montevideo | 103716 | Blood | 13 | 25 |  |  |
| *S.* Montevideo | 92608 | Blood | 1 |  |  |  |
| *S.* Montevideo | 96116 | Blood | 1 |  |  |  |
| *S.* Montevideo | 105802 | Blood | 28 |  |  |  |
| *S.* Montevideo | 115434 | Blood | 4 |  |  |  |
| *S.* Montevideo | 126972 | Blood | 1 |  |  |  |
| *S.* Montevideo | 134623 | Blood | 1 |  |  |  |
| *S.* Montevideo | 125919 | Stool | 1 | 26 |  |  |
| *S.* Montevideo | 111711 | Stool | 35 |  |  |  |
| *S.* Montevideo | 112357 | Stool | 32 |  |  |  |
| *S.* Montevideo | 112258 | Stool | 31 |  |  |  |
| *S.* Montevideo | 117459 | Stool | 25 |  |  |  |
| *S.* Montevideo | 118252 | Stool | 38 |  |  |  |
| *S.* Montevideo | 119948 | Stool | 1 |  |  |  |
| *S.* Montevideo | 120310 | Stool | 7 |  |  |  |
| *S.* Montevideo | SARB30 | SGSC | NA |  |  |  |
| *S.* Montevideo | SARB31 | SGSC | NA |  |  |  |
| *S.* Newport | 91532 | Blood | 2 | 27 | + | + |
| *S.* Newport | 125047 | Stool | 37 | 28 |  |  |
| *S.* Typhimurium | 116449 | Blood | 56 | 29 | + |  |
| *S.* Typhimurium | 98666 | Blood | 54 | 30 | + |  |
| *S.* Typhimurium | 103259 | Blood | 49 | 31 | + | + |
| *S.* Typhimurium | 93561 | Stool | 12 | 32 | + |  |
| *S.* Typhimurium | 78651 | Stool | 1 |  |  |  |
| *S.* Typhimurium | 82788 | Stool | 1 |  |  |  |
| *S.* Typhimurium | 88359 | Stool | 38 |  | + |  |
| *S.* Typhimurium | 92273 | Stool | <1 |  |  |  |
| *S.* Typhimurium | 93130 | Blood | 56 |  | + |  |
| *S.* Typhimurium | 96452 | Blood | 69 |  |  |  |
| *S.* Typhimurium | 98001 | Stool | 47 |  | + |  |
| *S.* Typhimurium | 99958 | Blood | <1 |  |  |  |
| *S.* Typhimurium | 103400 | Stool | <1 |  |  |  |
| *S.* Typhimurium | 104768 | Blood | 74 |  |  |  |
| *S.* Typhimurium | 109074 | Blood | 1 |  |  |  |
| *S.* Typhimurium | 109971 | Blood | 1 |  |  |  |
| *S.* Typhimurium | 110128 | Blood | 81 |  |  |  |
| *S.* Typhimurium | 111682 | Blood | 6 |  | + |  |
| *S.* Typhimurium | 112360 | Blood | 62 |  |  |  |
| *S.* Typhimurium | 113279 | Blood | 70 |  |  |  |
| *S.* Typhimurium | 114745 | Blood | 85 |  |  |  |
| *S.* Typhimurium | 115026 | Blood | 93 |  |  |  |
| *S.* Typhimurium | 115043 | Stool | 14 |  | + |  |
| *S.* Typhimurium | 115477 | Stool | 2 |  |  |  |
| *S.* Typhimurium | 117507 | Stool | 79 |  |  |  |
| *S.* Typhimurium | 124652 | Stool | <1 |  |  |  |
| *S.* Typhimurium | 127587 | Stool | <1 |  |  |  |
| *S.* Typhimurium | 129307 | Stool | <1 |  |  |  |
| *S.* Typhimurium | 130100 | Stool | 3 |  | + |  |
| *S.* Typhimurium | 133150 | Stool | <1 |  |  |  |
| *S.* Typhimurium | LT2 | Lab stock | NA |  |  |  |
| *S.* Typhimurium | SL1344 | Lab stock | NA |  |  |  |
| *S.* Typhimurium | DT104L | SGSC | NA |  |  |  |
| *S.* Typhimurium | 14028s | SGSC | NA |  |  |  |
| *S.* Virchow | 102372 | Blood | 4 | 33 |  |  |
| *S.* Virchow | 103033 | Blood | 9 | 34 | + | + |
| *S.* Virchow | 125755 | Stool | 6 | 35 |  |  |
