# Supplementary material for: Virulence Gene Profiling and Pathogenicity Characterization of Non-Typhoidal Salmonella Accounted for Invasive Disease in Humans
Source: PLoS One. 2013 Mar 7;8(3):e58449. doi: 10.1371/journal.pone.0058449 (PMC3591323; doi:10.1371/journal.pone.0058449)
Supplement: Table S2 — Primers used in this study. (DOC) [file pone.0058449.s002.doc]

**Table S2 Primers used in this study.**

| Primer | Sequence (5' to 3') | | Use |
| --- | --- | --- | --- |
|  | Forward | Reverse |  |
| spi7vex | [CGACATTTTTCCTGCTTTCG](../../../../V:%5CJotham%5Cמחקר%5CHandy%20tools%20&amp%3B%20lists%5Cרצפים%5CPrimers%5CSPI-7%20vexA%20primers.txt) | [ATGCGGCTTTCACCTTAGC](../../../../V:%5CJotham%5Cמחקר%5CHandy%20tools%20&amp%3B%20lists%5Cרצפים%5CPrimers%5CSPI-7%20vexA%20primers.txt) | Southern blot |
| spi8 | [AAATCAGGTAAGGCATCAAAGG](../../../../V:%5CJotham%5Cמחקר%5CHandy%20tools%20&amp%3B%20lists%5Cרצפים%5CPrimers%5CSPI-8%20primers.txt) | [CTCAGGTGTTCCATCACTTTCC](../../../../V:%5CJotham%5Cמחקר%5CHandy%20tools%20&amp%3B%20lists%5Cרצפים%5CPrimers%5CSPI-8%20primers.txt) | Southern blot |
| spi10sef | [TTCATTGTCTCTGTTTTTCTGATTG](../../../../V:%5CJotham%5Cמחקר%5CHandy%20tools%20&amp%3B%20lists%5Cרצפים%5CPrimers%5CSPI-10%20sefD.txt) | [TTAAATAGGTATCAACGGGAATG](../../../../V:%5CJotham%5Cמחקר%5CHandy%20tools%20&amp%3B%20lists%5Cרצפים%5CPrimers%5CSPI-10%20sefD.txt) | Southern blot |
| spi15 | [CTTGCACCTTTATCATCACTGG](../../../../V:%5CJotham%5Cמחקר%5CHandy%20tools%20&amp%3B%20lists%5Cרצפים%5CPrimers%5CSPI-15.txt) | [AGTGTCTCCCTCCTGAACTGC](../../../../V:%5CJotham%5Cמחקר%5CHandy%20tools%20&amp%3B%20lists%5Cרצפים%5CPrimers%5CSPI-15.txt) | Southern blot |
| spi17 | GCAATAGCGGTTTTATCTGTGG | [GTTAAATGTCCCCAATCAACG](../../../../V:%5CJotham%5Cמחקר%5CHandy%20tools%20&amp%3B%20lists%5Cרצפים%5CPrimers%5CSPI-17.txt) | Southern blot |
| ST64b | [ATGGGCGCTATTGTCTTAACG](../../../../V:%5CJotham%5Cמחקר%5CHandy%20tools%20&amp%3B%20lists%5Cרצפים%5CPrimers%5CST64b.txt) | [AGCTCAGGGAATTTGGATTCG](../../../../V:%5CJotham%5Cמחקר%5CHandy%20tools%20&amp%3B%20lists%5Cרצפים%5CPrimers%5CST64b.txt) | Southern blot |
| sgi1 | TTTCGCCAATCGAATAATCC | ACTTGAACCCAATGCTCTGC | Southern blot |
| hpi1 | [GACCTGACCTGGCATTTAACC](../../../../V:%5CJotham%5Cמחקר%5CHandy%20tools%20&amp%3B%20lists%5Cרצפים%5CPrimers%5CHPI-1.txt) | [GCATTGCTTAATGTCTGCATCC](../../../../V:%5CJotham%5Cמחקר%5CHandy%20tools%20&amp%3B%20lists%5Cרצפים%5CPrimers%5CHPI-1.txt) | Southern blot |
| ssek3 | TATCAATCTCAAATCATGG | CGCGTTTATATCATACGTTTGC | PCR |
| sspH1 | GGTCACAGGACACGTTCTACG | GCGCTTCTTCGTAATTTTCC | PCR |
| sopE | CATAGCGCCTTTTCTTCAGG | ATGCCTGCTGATGTTGATTG | PCR |
| pefA | TAAGCCACTGCGAAAGATGC | GCGTGAACTCCAAAAACCCG | PCR |
| sodC | ATGACACCACAGGCAAAACG | AGATGAACGATGCCCTGTCC | PCR |
| sseI | CGCCATCATCAGTAACCGCC | CTGCTGACCACATCCTCCC | PCR |
| STM2759 | ACCATTTTCACCTGGGCTCC | CGTTCAGGTTTTGTCGCTGG | PCR |
| gatC | ATTGGTATCGGCTTCGTGGG | ATCCCCAGCCAGTATGAACC | PCR |
| gogB | ACGAGGCGACATCAAACCTT | GACCGTTCCCTCAATCGTGT | PCR |
| tcfA | TCGCTATGTTTGCATGTGGT | TTCAGGAACAGCCTCGAAGT | PCR |
| hlyE | GCGTGATTGAAGGGAAATTG | CGAAAAGCGTCTTCTTACCG | PCR |
| cdtB | CACTCGGCTATTGATGTTGG | ATTTGCGTGGGTTCTGTAGG | PCR |
| tcfA-RT | aggaggtaccagcagggaat | ttcaggaacagcctcgaagt | RT-PCR |
| hlyE-RT | gcagcaattggggagataaa | cgagaagcgtcttcttaccg | RT-PCR |
| taiA-RT | TGAACCCAAACCTGTTGTGA | GCAACATAAATTCGCTAATCTC | RT-PCR |
| cdtB-RT | atttgcgtgggttctgtagg | ggatgctgcagctattgtca | RT-PCR |
| Clone-Tcf | TTTTCTAGAGCTGGAAGCTGAAGTCGAAC | TTTGAGCTCACCGCACGAACAATGATGT | PCR *tcfA-tioA* |
| Clone-Cdt | TTTTCTAGAACAAATAAACCAGGCGCTTA | TTTGAGCTCTCAACGTCATGAAACAATGG | PCR: *cdtB-sty1891* |
| Clone-SPI18 | TTTTCTAGAGCCGGGTTTTGATATCAGTT | TTTGAGCTCACACGGAGCGAATGAAAAAG | PCR *hlyE-taiA* |
