# Supplementary material for: Virulence Gene Profiling and Pathogenicity Characterization of Non-Typhoidal Salmonella Accounted for Invasive Disease in Humans
Source: PLoS One. 2013 Mar 7;8(3):e58449. doi: 10.1371/journal.pone.0058449 (PMC3591323; doi:10.1371/journal.pone.0058449)
Supplement: Table S4 — Novel genomic islets identified in this study. Discrete regions with variable presence among the 12 NTS serovars are listed. Plus (+) indicates the presence of the islets, minus sign (-) indicates its absence and plus-minus sign (±) indicates partial or mosaic presence. (DOC) [file pone.0058449.s004.doc]

| # | Start | End | Number of ORFs | size (kb) | %  GC | Function | Typhimurium |  | Schwarzengrund | 9,12;I,v;- | Bredeney | Choleraesuis | Dublin | Enteritidis | Hadar | Heidelberg | Montevideo | Newport | Virchow |
| --- | --- | --- | --- | --- | --- | --- | --- | --- | --- | --- | --- | --- | --- | --- | --- | --- | --- | --- | --- |
| 1 | STM0409 | STM0410 | 2 | 1.08 | 52 | igeR regulator of Cdt, flagellar & SPI-1 genes | + |  | - | - | - | + | + | + | + | + | - | + | + |
| 2 | STM0437 | STM0438 | 2 | 3.02 | 42 | chick colonization attenuation (STM0438) | + |  | - | - | - | + | + | + | + | + | - | + | + |
| 3 | STM0654 | STM0660 | 8 | 8.45 | 52 | *ybe* operon | + |  | - | - | - | + | + | + | + | + | - | + | + |
| 4 | STM0839 | STM0840 | 2 | 1.49 | 45.5 | hydrolase (ybiV(2)), inner membrane protein | + |  | - | - | - | - | + | + | + | - | - | - | + |
| 5 | STM1001 | STM1003 | 3 | 3.67 | 46 | diaminopropionate ammonia lyase, leucine response regulator | + |  | - | - | - | + | + | + | + | + | - | + | + |
| 6 | SEN2001 | SEN2005 | 5 | 6 | 48 | endoprotease, TPR repeat protein | + |  | - | - | - | + | + | + | + | + | - | + | + |
| 7 | STM2289 | STM2292 | 4 | 4.18 | 53 | yfa operon | + |  | + | - | + | + | + | + | + | + | + | + | + |
| 8 | STM3169 | STM3171 | 3 | 2.83 | 51 | mice virulence, intramacrophage growth | + |  | - | - | - | + | + | + | + | + | - | + | + |
| 9 | STM3528 | STM3533 | 6 | 8.5 | 51 | STM3529 hilA activation | + |  | - | - | - | + | + | + | + | + | + | + | + |
| 10 | STM3558 | STM3559 | 2 | 0.59 | 53 | homology to death -on-curing protein of phage P1 | + |  | - | + | - | + | + | + | + | - | + | + | + |
| 11 | STM3651 | STM3653 | 3 | 1.41 | 45 | acetyltransferases | + |  | + | ± | + | + | + | + | ± | + | - | - | ± |
| 12 | STM3696 | STM3698 | 3 | 3.9 | 51 | mandelate racemase / muconate lactonizing enzyme, permease | + |  | - | - | - | + | + | + | + | + | - | + | + |
| 13 | STM4389 | STM4390 | 2 | 0.68 | 47 |  | + |  | - | - | - | + | + | + | + | + | - | + | + |
| 14 | SEN0163 | SEN0164 | 2 | 0.88 | 44 |  | - |  | - | - | - | + | + | + | + | + | - | + | + |
| 15 | SPA3794 | SPA3795 | 2 | 0.85 | 33 |  | - |  | - | + | - | + | + | + | + | + | - | + | + |
| 16 | STY3644 | STY3645 | 2 | 1.2 | 54 | effector domain of the CAP family of transcription factors | - |  | + | - | + | - | - | - | - | - | + | - | - |

**Table S4 Genomic islets identified in this study.**
