# Supplementary material for: Virulence Gene Profiling and Pathogenicity Characterization of Non-Typhoidal Salmonella Accounted for Invasive Disease in Humans
Source: PLoS One. 2013 Mar 7;8(3):e58449. doi: 10.1371/journal.pone.0058449 (PMC3591323; doi:10.1371/journal.pone.0058449)
Supplement: Table S6 — Distribution of pefA, sodC, sseI, STM2759, gatC and gogB among invasive and enteritis isolates of S. Typhimurium. The presence of pefA, sodC, sseI, STM 2759, gatC and gogB was examined by PCR in 15 blood and 15 stool isolates of S. Typhimurium. The primers used for this analysis are listed in Table S2. A “+” sign indicates gene presence and “–“ sign indicates its absence. (DOCX) [file pone.0058449.s006.docx]

**Table S6 Distribution of *pefA, sodC, sseI,* STM 2759*, gatC* and *gogB* among invasive and enteritis isolates of *S.* Typhimurium.**

|  | **Isolate** | **Target locus** | | | | | |
| --- | --- | --- | --- | --- | --- | --- | --- |
|  |  | ***pefA*** | ***sodC*** | ***sseI*** | ***stm2759*** | ***gatC*** | ***gogB*** |
| **Invasive isolates** | 93130 | *+* | + | *+* | + | + | + |
|  | 96452 | *-* | *+* | *+* | + | + | + |
|  | 98666 | *+* | + | *+* | + | + | + |
|  | 99958 | *+* | *+* | *+* | + | + | - |
|  | 103259 | *+* | + | *+* | + | + | + |
|  | 104768 | *+* | *+* | *+* | + | + | + |
|  | 109074 | *+* | + | *+* | + | + | + |
|  | 109971 | *+* | + | *+* | + | + | + |
|  | 110128 | *+* | + | *+* | + | + | + |
|  | 111682 | *+* | + | *+* | + | + | + |
|  | 112360 | *-* | *-* | *+* | + | + | - |
|  | 113279 | *+* | - | *+* | + | + | - |
|  | 114745 | *+* | + | *+* | + | + | + |
|  | 115026 | *+* | + | *+* | + | + | + |
|  | 116449 | *+* | + | *+* | + | + | + |
|  | **Total positive** | **13/15 (86.6%)** | **13/15 (86.6%)** | **15/15 (100%)** | **15/15 (100%)** | **15/15 (100%)** | **12/15 (80%)** |
| **Enteritis isolates** | 78651 | + | + | *+* | + | + | - |
|  | 82788 | + | + | *+* | + | + | + |
|  | 92273 | + | + | *+* | + | + | + |
|  | 103400 | - | - | *+* | + | + | - |
|  | 115477 | + | + | *+* | + | + | + |
|  | 117507 | + | + | *+* | + | + | + |
|  | 124652 | - | + | *+* | + | + | + |
|  | 127587 | + | + | *+* | + | + | + |
|  | 129307 | - | + | *+* | + | + | + |
|  | 93561 | + | + | + | + | + | + |
|  | 88359 | + | - | - | + | + | - |
|  | 98001 | + | + | + | - | - | + |
|  | 130100 | - | + | + | + | + | + |
|  | 115043 | + | + | + | + | + | + |
|  | 133150 | + | + | *+* | + | + | + |
|  | **Total positive** | **11/15 (73.3%)** | **13/15 (86.6%)** | **14/15 (93.3%)** | **14/15 (93.3%)** | **14/15 (93.3%)** | **12/15 (80%)** |
