# Supplementary material for: Virulence Gene Profiling and Pathogenicity Characterization of Non-Typhoidal Salmonella Accounted for Invasive Disease in Humans
Source: PLoS One. 2013 Mar 7;8(3):e58449. doi: 10.1371/journal.pone.0058449 (PMC3591323; doi:10.1371/journal.pone.0058449)
Supplement: Table S7 — Distribution of cdtB, hlyE, and tcfA among invasive and enteritis isolates of serovars Schwarzengrund, 9,12:l,v:-, Bredeney and Montevideo. The presence of cdtB, hlyE, and tcfA was examined by PCR in clinical isolates of serovars Schwarzengrund, 9,12:l,v:-, Bredeney and Montevideo from blood (invasive) and stool (gastroenteritis) sources. The primers used for this analysis are listed in Table S2. A “+” sign indicates gene presence and “–“ sign indicates its absence. (DOC) [file pone.0058449.s007.doc]

**Table S7 Distribution of *cdtB, hlyE*, and *tcfA* among invasive and enteritis isolates of serovars Schwarzengrund, 9,12:l,v:-, Bredeney and Montevideo.**

|  | **Schwarzengrund** | | | |  | **9,12:l,v:-** | | | | |
| --- | --- | --- | --- | --- | --- | --- | --- | --- | --- | --- |
|  | **Isolate** | ***cdtB*** | ***hlyE*** | ***tcfA*** |  | **Invasive isolates** | **Isolate** | ***cdtB*** | ***hlyE*** | ***tcfA*** |
| **Invasive isolates** | 124986 | + | + | + |  |  | 78639 | + | + | + |
|  | 124983 | + | + | + |  |  | 96114 | + | + | + |
|  | 124985 | + | + | + |  |  | 94293 | + | + | + |
|  | 131982 | + | + | - |  |  | 92027 | + | + | + |
|  | Total positive | 4/4 (100%) | 4/4 (100%) | 3/4 (75%) |  |  | 92287 | + | + | + |
| **Enteritis isolates** | 123249 | + | + | + |  |  | 96659 | + | + | + |
|  | 91899 | + | + | - |  |  | 99366 | + | + | + |
|  | 93610 | + | + | - |  |  | 101823 | + | + | + |
|  | 95798 | + | + | - |  |  | 103439 | + | + | + |
|  | 96750 | + | + | - |  |  | 111027 | + | + | + |
|  | 109107 | + | + | - |  |  | 121750 | + | + | + |
|  | 110382 | + | + | - |  |  | 125271 | + | + | + |
|  | 110460 | + | + | - |  |  | 125377 | + | + | + |
|  | 112399 | + | + | - |  |  | 133986 | + | + | + |
|  | 112525 | + | + | - |  |  | 134055 | + | + | + |
|  | 112900 | + | + | - |  |  | **Total positive** | **15/15 (100%)** | **15/15 (100%)** | **15/15 (100%)** |
|  | 118194 | + | + | - |  | **Enteritis isolates** | 125936 | + | + | + |
|  | **Total positive** | **12/12 (100%)** | **12/12 (100%)** | **1/12 (8.33%)** |  |  | 95135 | + | + | + |
|  |  |  |  |  |  |  | 96735 | + | + | + |
| **Bredeney** | | | | |  |  | 97604 | + | + | + |
| **Invasive isolates** | **Isolate** | ***cdtB*** | ***hlyE*** | ***tcfA*** |  |  | 106208 | + | + | + |
|  | 96115 | + | + | + |  |  | 106281 | + | + | + |
|  | 90321 | + | + | + |  |  | 110695 | + | + | + |
|  | 92025 | + | + | + |  |  | 111449 | + | + | + |
|  | 92339 | + | + | + |  |  | 128109 | + | + | + |
|  | 111361 | + | + | + |  |  | 131348 | + | + | + |
|  | 117665 | + | + | + |  |  | 132667 | + | + | + |
|  | 119214 | + | + | + |  |  | 117808 | + | + | + |
|  | 123632 | + | + | + |  |  | 127595 | + | + | + |
|  | 123896 | + | + | + |  |  | **Total positive** | **13/13 (100%)** | **13/13 (100%)** | **13/13 (100%)** |
|  | 128407 | + | + | + |  |  |  |  |  |  |
|  | 130875 | + | + | + |  | **Montevideo** | | | | |
|  | 133975 | + | + | + |  | **Invasive isolates** | **Isolate** | ***cdtB*** | ***hlyE*** | ***tcfA*** |
|  | 134592 | + | + | + |  |  | 111072 | + | + | + |
|  | **Total positive** | **13/13 (100%)** | **13/13 (100%)** | **13/13 (100%)** |  |  | 103716 | + | + | + |
| **Enteritis isolates** | 125816 | + | + | + |  |  | 92608 | + | + | + |
|  | 93520 | + | + | + |  |  | 96116 | + | + | + |
|  | 95097 | + | + | + |  |  | 105802 | + | + | + |
|  | 97597 | + | + | + |  |  | 115434 | + | + | + |
|  | 99441 | + | + | + |  |  | 126972 | + | + | + |
|  | 101983 | + | + | + |  |  | 134623 | + | + | + |
|  | 113272 | + | + | + |  |  | **Total positive** | **8/8 (100%)** | **8/8 (100%)** | **8/8 (100%)** |
|  | 119210 | + | + | + |  | **Enteritis isolates** | 125919 | + | + | + |
|  | 121976 | + | + | + |  |  | 112357 | + | + | + |
|  | 127708 | + | + | + |  |  | 112258 | + | + | + |
|  | 130076 | + | + | + |  |  | 117459 | + | + | + |
|  | 131122 | + | + | + |  |  | 119948 | + | + | + |
|  | Total positive | 12/12 (100%) | 12/12 (100%) | 12/12 (100%) |  |  | 120310 | + | + | + |
|  |  |  |  |  |  |  | Total positive | 6/6 (100%) | 6/6 (100%) | 6/6 (100%) |
