# Supplementary material for: Virulence Gene Profiling and Pathogenicity Characterization of Non-Typhoidal Salmonella Accounted for Invasive Disease in Humans
Source: PLoS One. 2013 Mar 7;8(3):e58449. doi: 10.1371/journal.pone.0058449 (PMC3591323; doi:10.1371/journal.pone.0058449)
Supplement: Text S1 — Presence of the spv operon and the virulence plasmid; Prophage and bacteriophage remnants; Identification of novel genomics islets. (DOCX) [file pone.0058449.s008.docx]

**Text S1**

**Presence of the *spv* operon and the virulence plasmid**

One of the main virulence factors that have been suggested to affect *Salmonella* manifestation is the *spv* operon (*spvABCDR*) [[1](#_ENREF_1)]. This locus encodes the SpvB toxin, which was shown to enhance *S*. Typhimurium pathogenicity in mice [[2](#_ENREF_2),[3](#_ENREF_3)] and was proved to be necessary for systemic disease by *S*. Dublin in calves [[4](#_ENREF_4)]. The *spv* locus is known to be present on the virulence plasmid of certain subspecies I serovars, but was also identified on the chromosome of strains belong to subspecies II, IIIa, IV, and VII [[5](#_ENREF_5)]. Fierer *et al*. have shown that *S*. Typhimurium isolates that lack the *spv* genes are less able to proliferate beyond the superficial epithelial layer and therefore these isolates tend to cause gastroenteritis, and are less associated with systemic disease [[6](#_ENREF_6)]. Therefore, it was concluded that the *spv* operon is required for systemic disease in NTS serovars [[5](#_ENREF_5)]. Our microarray data indicated that among the 12 serovars tested, only serovars known to possess the virulence plasmid (i.e. Choleraesuis, Dublin, Enteritidis, and Typhimurium) harbored this locus (Table 1), suggesting that the role of *spv* is most-likely serovar-specific and it is not required by all NTS serovars to cause extra-intestinal infection.

**Prophage and bacteriophage remnants**

*Salmonella* genomes were shown to contain different temperate prophages or phage remnants [[7](#_ENREF_7)]. Occasionally, these elements carry genes directly involved in virulence including superoxide dismutase SodCI and SodCIII systems (encoded in Gifsy-2 and Fels-1, respectively) and several T3SS effectors [[8](#_ENREF_8)]. CGH analysis examined the presence of 16 phages-associated regions showed sporadic and mosaic distribution across the bacteremic isolates (Table 1). Gifsy-3 (encodes PagJ and the SspH1 effectors) and Fels-1 (encodes the Cu/Zn superoxide dismutase SodCIII) were absent from all strains. Gifsy-2 (encodes SodCI, GvrA, SseI, GtgE) was missing in six isolates (Schwarzengrund, 9,12:l,v:-, Bredeney, Hadar, Montevideo, and Virchow). Three isolates (Montevideo, Schwarzengrund, and 9,12:l,v:-) were found to lack 13/16 phage-originated regions examined (sopEФ, Gifsy 1, Gifsy 2, Gifsy 3, Fels1, Fels 2, Def4, ST10, ST15, ST18, ST27, ST35, and ST64b). Prophage genes encoded in the STM4195-4219 (Def4) region [[9](#_ENREF_9)] were missing or divergent in 8 isolates (Hadar, Bredeney, Montevideo, 9,12:l,v:-, Dublin, Schwarzengrund, Enteritidis, and Newport). Two known *S*. Typhi CT18 prophages elements (STY1048-STY1073 and STY2013-STY2039 [[10](#_ENREF_10)]) were identified in the *S.* Choleraesuis isolate and another *S*. Typhi homologous phage (ST35) was found in the invasive *S.* Dublin isolate. These results further exemplify the contribution of bacteriophage genes as a mean of genetic diversification, but also indicate that several phage-encoded virulence factors identified in *S*. Typhimurium including SopE, SodCIII and SspH1 [[11](#_ENREF_11)] are unnecessary for invasive disease manifestation in humans.

**Identification of novel genomics islets**

In addition to the already known genomic islands and prophages represented on the array, CGH facilitated the identification of 16 distinct regions with clear variable presence across the serovars, which were not reported previously as GIs (Table S4). These islets are expected to be involved in metabolic pathways (STM0839-STM0840, STM2289-STM2292, STM3528-STM3533, STM3651-STM3653, STM3696-3698); stress response (STM0654-STM0660); gene regulation (STM0409-STM0410) and virulence (STM0437-STM0438 [[12](#_ENREF_12)], STM3169-STM3171 [[13](#_ENREF_13)]). Genes encoded on these islets may play a role in host or niche adaptation, but are serovar-specific and not generally required for systemic infection, as they were present only in a subset of the iNTS strains.

**References**

1. Rotger R, Casadesus J (1999) The virulence plasmids of *Salmonella*. Int Microbiol 2: 177-184.

2. Gulig PA, Doyle TJ, Hughes JA, Matsui H (1998) Analysis of host cells associated with the Spv-mediated increased intracellular growth rate of Salmonella typhimurium in mice. Infect Immun 66: 2471-2485.

3. Lesnick ML, Reiner NE, Fierer J, Guiney DG (2001) The *Salmonella* *spvB* virulence gene encodes an enzyme that ADP-ribosylates actin and destabilizes the cytoskeleton of eukaryotic cells. Mol Microbiol 39: 1464-1470.

4. Libby SJ, Adams LG, Ficht TA, Allen C, Whitford HA, et al. (1997) The *spv* genes on the *Salmonella dublin* virulence plasmid are required for severe enteritis and systemic infection in the natural host. Infect Immun 65: 1786-1792.

5. Boyd EF, Hartl DL (1998) *Salmonella* virulence plasmid. Modular acquisition of the *spv* virulence region by an F-plasmid in *Salmonella enterica* subspecies I and insertion into the chromosome of subspecies II, IIIa, IV and VII isolates. Genetics 149: 1183-1190.

6. Fierer J, Krause M, Tauxe R, Guiney D (1992) *Salmonella typhimurium* bacteremia: association with the virulence plasmid. J Infect Dis 166: 639-642.

7. Vernikos GS, Thomson NR, Parkhill J (2007) Genetic flux over time in the *Salmonella* lineage. Genome Biol 8: R100.

8. Ehrbar K, Hardt WD (2005) Bacteriophage-encoded type III effectors in *Salmonella enterica* subspecies 1 serovar Typhimurium. Infect Genet Evol 5: 1-9.

9. McClelland M, Sanderson KE, Spieth J, Clifton SW, Latreille P, et al. (2001) Complete genome sequence of *Salmonella enterica* serovar Typhimurium LT2. Nature 413: 852-856.

10. Deng W, Liou SR, Plunkett G, 3rd, Mayhew GF, Rose DJ, et al. (2003) Comparative genomics of *Salmonella enterica* serovar Typhi strains Ty2 and CT18. J Bacteriol 185: 2330-2337.

11. Figueroa-Bossi N, Uzzau S, Maloriol D, Bossi L (2001) Variable assortment of prophages provides a transferable repertoire of pathogenic determinants in *Salmonella*. Mol Microbiol 39: 260-271.

12. Morgan E, Campbell JD, Rowe SC, Bispham J, Stevens MP, et al. (2004) Identification of host-specific colonization factors of *Salmonella enterica* serovar Typhimurium. Mol Microbiol 54: 994-1010.

13. Chaudhuri RR, Peters SE, Pleasance SJ, Northen H, Willers C, et al. (2009) Comprehensive identification of *Salmonella enterica* serovar typhimurium genes required for infection of BALB/c mice. PLoS Pathog 5: e1000529.
